# Integrating viral kinetics and population spread in a one health framework to explain variant-specific epidemic dynamics^☆^

**DOI:** 10.1016/j.onehlt.2026.101389

**Published:** 2026-03-17

**Authors:** Hyosun Lee, Byul Nim Kim, Sunmi Lee

**Affiliations:** Department of Applied Mathematics, Kyung Hee University, Yongin, 17104, South Korea

**Keywords:** Multi-scale epidemic modeling, Within-host viral kinetics, Agent-based model (ABM), Variant-specific transmission, Data-integrated One Health approach, Risk assessment for emerging viruses

## Abstract

The COVID-19 pandemic underscored the importance of modeling frameworks that integrate biological mechanisms with heterogeneous social contact patterns to accurately characterize variant-specific transmission. Motivated by a One Health perspective that connects human infection biology, behavioral dynamics, and environmental transmission factors, we present a data-integrated and mechanistic approach designed to support proactive risk assessment and public-health preparedness. While classical compartmental models offer essential baseline insight, their simplifying assumptions limit the representation of time-varying infectiousness and realistic transmission heterogeneity. We introduce a multi-scale agent-based model that links empirically inferred SARS-CoV-2 viral kinetics to population-level spread through a mechanistic mapping from viral load to infection probability. Ct trajectories are estimated using hierarchical Bayesian inference and incorporated into a structured contact network, enabling coupling of within-host viral dynamics with social interaction patterns. This One Health-aligned modeling architecture supports rigorous data integration and biologically grounded estimation of variant-specific epidemic behavior. Our results demonstrate that differences in viral kinetics substantially reshape epidemic trajectories. Variants with rapid viral expansion and short infectious periods produce earlier and sharper peaks, whereas slower proliferation and prolonged clearance lead to delayed yet larger outbreaks. Incorporating time-varying infectiousness also generates heterogeneous secondary-case distributions and occasional high-impact transmission events without imposing ad-hoc superspreading parameters, highlighting biological drivers of overdispersion. By linking within-host viral dynamics to network-level transmission, this framework provides a scalable tool for variant surveillance, quantitative risk assessment, and timing-sensitive intervention planning. It can be extended to environmentally mediated pathogens, strengthening One Health-oriented data integration and epidemic estimation for future emerging threats.

## 1 Introduction

The COVID-19 pandemic underscored the need for modeling frameworks that integrate biological mechanisms with social structure to explain and estimate infectious disease spread. Since the World Health Organization declared COVID-19 a global pandemic on 11 March 2020 [1], and national authorities such as Korea’s KDCA implemented extensive surveillance and containment programs [2], quantitative models have supported policy decisions at every stage. Classical compartmental models remain foundational, yet mean-field assumptions can obscure heterogeneity in contact patterns, behavioral adaptation, and within-host processes, potentially biasing estimates and limiting targeted, resource-efficient intervention design. Because transmission emerges from mechanisms operating across molecular, cellular, individual, and population scales, integrative approaches are increasingly required to capture cross-scale interactions [3], [4]. Alongside the demand for operational decision-support, there is a conceptual need to align biological realism with policy levers so mechanistic insights translate into actionable control strategies.

We adopt an integrated approach aligned with One Health priorities, which recognize interdependencies among biological processes, human behavior, and environmental transmission pathways. This perspective motivates data-integrated, mechanistic models that connect molecular infection dynamics to public-health decision making. Models that bridge within-host viral kinetics, individual variation, and population mixing can strengthen surveillance, preparedness, and intervention planning.

Agent-based models (ABMs) naturally represent these complexities by simulating individuals in multilayer contact networks and mobility patterns, capturing behavioral heterogeneity, network topology, and stochastic exposure. This structure enables evaluation of targeted strategies (e.g., hub-focused distancing, ring testing, setting-specific controls) and has supported mobility-informed reopening analyses [5] and integrated testing–vaccination–tracing evaluations [6]. However, many ABMs assume constant or ad hoc transmissibility, overlooking within-host replication, immunity, and time-varying infectiousness [7]. These omissions matter: epidemic tempo and peak burden are shaped by within-host dynamics [8], and overdispersed transmission demands a mechanistic treatment of heterogeneity and tail risk [9]. Multi-scale studies address this gap by embedding viral-load dynamics into transmission models: inoculum-dependent progression has been quantified [10]; stochastic and infection-age formulations link kinetics to contagion [11]; ABM extensions with viral-load–dependent infectiousness capture presymptomatic/asymptomatic spread [12], [13]; viral-trajectory–based approaches inform testing, vaccination, and antiviral policies [14], [15], [16]; evolutionary modeling suggests isolation may shape viral phenotypes [17]; and surveillance-calibrated ABMs incorporating kinetics better match observed patterns [18], [19]. Renewal-kernel theory further formalizes the connection between infection-age infectiousness and epidemic growth [20], [21]. Together, these advances motivate a unified, network-aware multi-scale framework.

Here we develop a biologically grounded, multi-scale ABM that derives temporal heterogeneity in infectiousness from variant-specific viral kinetics rather than imposing fixed profiles. Viral load is mapped to agent-level infection risk via a Hill function and propagated through heterogeneous contact networks. This construction captures (i) temporal heterogeneity from viral proliferation and clearance and (ii) population/network heterogeneity from contact structure, behavior, and stochastic exposure, enabling biologically grounded epidemic estimation at fixed $R_{0}$ and supporting evaluation of network-targeted interventions.

This study makes four contributions. First, we introduce a unified multi-scale ABM that maps viral load to infection risk via a Hill function, replacing ad hoc infectiousness profiles. Second, using empirically inferred, variant-specific trajectories, we show that modest replication/clearance differences reshape epidemic tempo and magnitude at fixed $R_{0}$—fast variants yield earlier, sharper peaks; slow-clearing ones, delayed, larger waves. Third, by isolating time-varying infectiousness, we quantify how temporal heterogeneity reallocates risk and, with network topology, synchronizes surges—effects homogeneous models miss. Finally, without added dispersion terms, the model reproduces long-tailed offspring distributions and rare high-impact events, revealing viral-kinetic drivers of superspreading. Together, these advances deliver a data-integrated, biologically grounded platform for surveillance, risk assessment, and timing-sensitive intervention design, and they show that even at fixed transmissibility, replication speed and clearance retime infectious contacts—rapid ascent accelerating peaks and prolonged clearance lengthening tails—through the interaction of within-host dynamics and social structure.

We present an ABM that explicitly embeds within-host viral dynamics into transmission simulations. By linking mechanistic viral replication to network-level spread, the framework bridges biological and social scales, supports data-integrated inference and risk assessment, and enables biologically grounded scenario analysis with targeted policy evaluation under fixed resources. This provides a rigorous foundation for preparedness against future respiratory-virus emergencies.

## 2 Data and methods

### 2.1 Data pre-processing for numerical simulations of viral load data

**Fig. 1:**
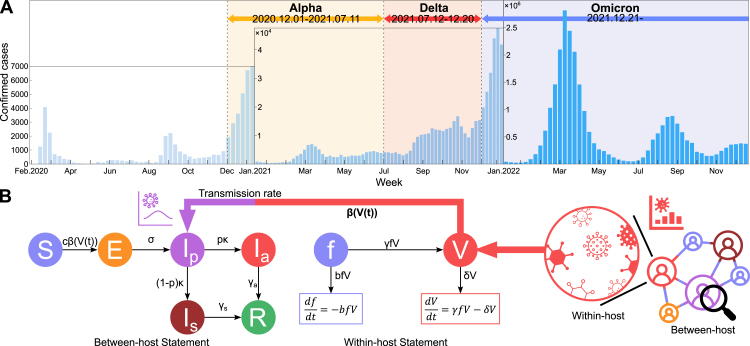
Overview of the simulation framework. A: Confirmed COVID-19 cases in Korea and variant dominance periods. B: SEIpIaIsR structure and coupling to within-host dynamics.

We anchored simulation settings to the early introduction windows of major SARS-CoV-2 variants in Korea (pre-variant: February 2020; Alpha: December 2020; Delta: July 2021; Omicron: January 2022). These periods correspond to distinct epidemic waves in national surveillance data (Fig. 1A) based on reports from the Korea Disease Control and Prevention Agency (KDCA) [2]. The longitudinal Ct measurements analyzed here are real (not synthetic) and come from the publicly available RT-qPCR dataset of Hay et al. [22], which includes repeated within-individual Ct observations with clinical metadata.

To estimate individual viral-load trajectories, we used publicly available longitudinal RT-qPCR cycle-threshold (Ct) data [22]. Each infection episode included repeated Ct values with metadata (variant, sampling day, vaccination status, antibody titer, symptom status). Observations were time-ordered to reconstruct trajectories spanning proliferation and clearance phases; episodes with insufficient sampling or minimal Ct variation were removed to ensure identifiable fits.

Inter-assay Ct calibration [22]:

(1) $$Ct^{\prime }=a_{0}+a_{1}Ct,\;\left(a_{0},a_{1}\right)=\left(-6.25,1.34\right).$$

Left-censoring at assay limit of detection (LOD):

(2) $$Ct^{\prime }\leftarrow \min\left\{Ct^{\prime },\mathrm{LOD}\right\},\;y=\min\left\{Ct^{\prime },\mathrm{LOD}\right\}\;\text{with left-censoring at LOD}.$$

Inclusion criteria to ensure informative trajectories:

(3) $$\min\left(Ct^{\prime }\right)\leq 32\;\text{and}\;\#\left\{\;Ct^{\prime }<\mathrm{LOD}\;\right\}\geq 3,$$

requiring at least three quantifiable points and sufficiently low Ct to capture viral ascent. Sampling times were expressed relative to first positive test and normalized to t=0 to align temporal scales across episodes and variants.

Host covariates were harmonized before inference. Vaccination status was fixed at detection, antibody titers aggregated into ordinal categories (highest value retained when multiple recorded), and symptom status reduced to a binary variable using a priority rule for inconsistent logs. Preprocessing steps (time normalization, censoring, covariate harmonization) were performed separately for each variant, producing standardized viral-trajectory datasets for hierarchical Bayesian inference and mechanistic within-host modeling.

### 2.2 Baseline ABM without within-host dynamics

The baseline agent-based model simulates SARS-CoV-2 transmission for variant m∈{Alpha,Delta,Omicron} over a static, undirected contact network (Fig. 1B). Simulations proceed in daily time steps for T=100 days, with each individual occupying one of six epidemiological states: susceptible (S), exposed (E), presymptomatic $\left(I_{p}\right)$, asymptomatic $\left(I_{a}\right)$, symptomatic $\left(I_{s}\right)$, or recovered (R). Susceptible individuals who contact infectious neighbors transition to the exposed class, after which symptomatic infections progress through $I_{p}\to I_{s}\to R$, while asymptomatic cases follow $I_{a}\to R$. The presymptomatic compartment is retained to represent early infectiousness and to ensure structural consistency with the multi-scale model. Additional methodological details are provided in Supplementary Information Section A.

Population dynamics evolve as:

(4) $$\Delta S=-S{\left[1-{\left(1-\beta_{p}^{\left(m\right)}\right)}^{\frac{\overset{¯}{k}I_{p}}{n}}{\left(1-\beta_{a}^{\left(m\right)}\right)}^{\frac{\overset{¯}{k}I_{a}}{n}}{\left(1-\beta_{s}^{\left(m\right)}\right)}^{\frac{\overset{¯}{k}I_{s}}{n}}\right]}^{\eta_{\mathrm{syn}}},$$

$$\Delta E=S{\left[1-{\left(1-\beta_{p}^{\left(m\right)}\right)}^{\frac{\overset{¯}{k}I_{p}}{n}}{\left(1-\beta_{a}^{\left(m\right)}\right)}^{\frac{\overset{¯}{k}I_{a}}{n}}{\left(1-\beta_{s}^{\left(m\right)}\right)}^{\frac{\overset{¯}{k}I_{s}}{n}}\right]}^{\eta_{\mathrm{syn}}}-\sigma^{\left(m\right)}E,$$

$$\Delta I_{p}=\sigma^{\left(m\right)}E-\kappa^{\left(m\right)}I_{p},$$

$$\Delta I_{a}=p^{\left(m\right)}\;\kappa^{\left(m\right)}I_{p}-\gamma_{a}^{\left(m\right)}I_{a},$$

$$\Delta I_{s}=\left(1-p^{\left(m\right)}\right)\kappa^{\left(m\right)}I_{p}-\gamma_{s}^{\left(m\right)}I_{s},$$

$$\Delta R=\gamma_{a}^{\left(m\right)}I_{a}+\gamma_{s}^{\left(m\right)}I_{s}.$$

The transition rates are defined as

(5) $$\sigma^{\left(m\right)}=\frac{1}{T_{E}^{\left(m\right)}},\;\kappa^{\left(m\right)}=\frac{1}{T_{p}^{\left(m\right)}},\;\gamma_{a}^{\left(m\right)}=\frac{1}{T_{a}^{\left(m\right)}},\;\gamma_{s}^{\left(m\right)}=\frac{1}{T_{s}^{\left(m\right)}}.$$

Stage-specific per-edge transmission probabilities are

(6) $$p_{i}^{\left(m\right)}=\left\{\begin{array}{cc} \beta_{p}^{\left(m\right)},\; & i\in I_{p},\\ \beta_{a}^{\left(m\right)}=\eta_{a}\beta_{p}^{\left(m\right)},\; & i\in I_{a},\\ \beta_{s}^{\left(m\right)}=\eta_{s}\beta_{p}^{\left(m\right)},\; & i\in I_{s}, \end{array}\right)\;\eta_{a}=0.2,\;\eta_{s}=0.5,$$

reflecting reduced asymptomatic and post-symptomatic transmission [23], [24], [25], [26].

Daily infection probability is computed via independent hazard union:

(7) $$P\left(S_{j}\to E_{j}\right)=1-\underset{i\in N_{j}^{\inf}}{\prod }\left(1-p_{i}^{\left(m\right)}\right).$$

To account for concurrent exposures, we apply a synergy adjustment:

(8) $$p_{\mathrm{tot}}^{\left(m\right)}=1-{\left(1-p_{\mathrm{union}}^{\left(m\right)}\right)}^{\eta_{\mathrm{syn}}},\;\eta_{\mathrm{syn}}\in \left(0,2\right].$$

Calibration to the target basic reproduction number $R_{0}$ is achieved by

(9) $$M^{\left(m\right)}=T_{p}^{\left(m\right)}+p^{\left(m\right)}\eta_{a}T_{a}^{\left(m\right)}+\left(1-p^{\left(m\right)}\right)\eta_{s}T_{s}^{\left(m\right)},\;\beta_{p}^{\left(m\right)}=\frac{R_{0}}{\overset{¯}{k}\;M^{\left(m\right)}}.$$

All state variables and coefficients appearing in Eq. (4) are summarized and defined in Table 1. Variant dependence enters through $\left\{\sigma^{\left(m\right)},\kappa^{\left(m\right)},\gamma_{a}^{\left(m\right)},\gamma_{s}^{\left(m\right)},p^{\left(m\right)},\beta_{p}^{\left(m\right)}\right\}$, while network properties $\left(n,\overset{¯}{k}\right)$ and synergy exponent $\eta_{\mathrm{syn}}$ are identical across variants.

To ensure variant-specific comparability, baseline transmission parameters are aligned to the temporal structure of infectiousness implied by within-host viral kinetics, while preserving a common target $R_{0}$ under an identical contact network. All population-level components are held fixed across variants, and variant dependence enters only through within-host kinetics and the corresponding alignment in the baseline model. Detailed formulations of the stage-wise alignment and $R_{0}$ calibration procedures are provided in Supplementary Information Section A.

**Table 1:** Summary of model parameters.

(a) Variant-specific between-host parameters (shared by Baseline & Multi-scale). Values are shown as mean (SD). Rate SDs were obtained using the delta-methodtransformation from duration-based estimates.					

Symbol (unit)	Meaning	Alpha	Delta	Omicron	Reference

$\sigma^{\left(m\right)}$ (day^−1^)	Transition rate from exposed E to presymptomatic infectious $I_{p}$	0.202(0.090)	0.227(0.127)	0.388(0.228)	[27], [28], [29]
$\kappa^{\left(m\right)}$ (day^−1^)	Transition rate from $I_{p}$ to symptomatic/asymptomatic stage	1.429(3.061)	1.563(3.662)	1.205(1.742)	[23], [30]
$p^{\left(m\right)}$ (-)	Probability of asymptomatic infection	0.220	0.142	0.324	[28], [31], [32], [33]
$\gamma_{a}^{\left(m\right)}$ (day^−1^)	Recovery rate of asymptomatic infectious individuals	0.075(0.011)	0.167(0.062)	0.167(0.103)	[22], [34]
$\gamma_{s}^{\left(m\right)}$ (day^−1^)	Recovery rate of symptomatic infectious individuals	0.075(0.011)	0.167(0.062)	0.167(0.103)	[22], [34]

(b) Common parameters (identical for all variants)	

Symbol	Meaning

n	Total population size
$\overset{¯}{k}$	Mean degree of contact network
$\eta_{a}$	Relative infectiousness of asymptomatic stage
$\eta_{s}$	Relative infectiousness of symptomatic stage
$\eta_{\mathrm{syn}}$	Synergy exponent for concurrent exposures

(c) Baseline-only parameters	

Symbol	Meaning

$\beta_{p}^{\left(m\right)}$	Presymptomatic transmission probability
$\beta_{a}^{\left(m\right)}$	Asymptomatic transmission probability
$\beta_{s}^{\left(m\right)}$	Symptomatic transmission probability

(d) Multi-scale-only parameters	

Symbol	Meaning

$b^{\left(m\right)}$	Target-cell infection rate (within-host ODE)
$\gamma^{\left(m\right)}$	Viral production rate
$\delta^{\left(m\right)}$	Viral clearance rate
$V_{0}^{\left(m\right)}$	Initial viral load
$\beta_{\max}^{\left(m\right)}$	Transmission scaling parameter

### 2.3 Multi-scale ABM with within-host viral dynamics

The multi-scale model couples variant-specific within-host viral kinetics to network transmission (Fig. 1B). For variant m∈{Alpha,Delta,Omicron}, each infected individual evolves viral load through a target-cell ODE system synchronized daily with the ABM time step.

Within-host dynamics used in the transmission simulations are generated by the target-cell–limited ODE system, which is numerically integrated for each infected individual and synchronized with the daily ABM time step. The resulting viral-load trajectories are then mapped to time-varying per-edge infection hazards through the Hill-type infectivity function and the scaling parameter $\beta_{\max}^{\left(m\right)}$. The piecewise continuous Ct formulation introduced elsewhere is used solely for inference and validation—summarizing longitudinal Ct data and estimating the ODE parameters—and does not enter the transmission simulations (see Supplementary Information Sections A and B). In our implementation, within-host parameters $\left(b^{\left(m\right)},\gamma^{\left(m\right)},\delta^{\left(m\right)},V_{0}^{\left(m\right)}\right)$ are drawn independently for each infected agent from the corresponding posterior distributions inferred from Ct data. This induces agent-specific viral load trajectories and introduces individual-level biological heterogeneity in viral replication and clearance dynamics. Heterogeneity in transmission therefore arises from both stochastic network interactions and posterior-informed variability in within-host kinetics.

**Fig. 2:**
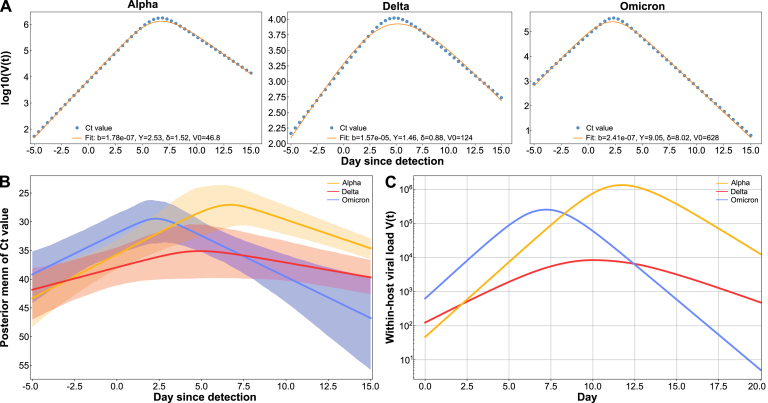
Estimated within-host parameters and fitted trajectories for each variant. **A** Robust estimates obtained by fitting the piecewise-linear Ct model and mapping to the ODE. **B** Piecewise-derived Ct curves with 95% posterior intervals. **C** ODE-simulated $\log_{10}V\left(t\right)$ trajectories using the estimated parameters.

Within-host dynamics are governed by

(10) $$\frac{df}{dt}=-b^{\left(m\right)}fV,\;\frac{dV}{dt}=\gamma^{\left(m\right)}fV-\delta^{\left(m\right)}V,\;{\left(f,V\right)|}_{t=0}=\left(1,V_{0}^{\left(m\right)}\right),$$

where $\left(b^{\left(m\right)},\gamma^{\left(m\right)},\delta^{\left(m\right)},V_{0}^{\left(m\right)}\right)$ are variant-specific parameters inferred from Ct data (Fig. 2).

Eq. (10) is a parsimonious target-cell–limited within-host model in which f(t)∈[0,1] denotes the fraction of susceptible target cells and V(t) the viral load. The parameters $b^{\left(m\right)}$, $\gamma^{\left(m\right)}$, and $\delta^{\left(m\right)}$ govern target-cell infection, viral production, and viral clearance, respectively, with initial condition $V\left(0\right)=V_{0}^{\left(m\right)}$. We use this minimal mechanistic structure to generate variant-specific, time-varying viral-load trajectories that are subsequently mapped to daily infectivity and per-edge transmission probabilities via the Hill-type coupling and $R_{0}$ calibration described below.

This formulation is derived from the classical target-cell–explicit infection virus (TEIV) framework under standard reduction assumptions. The term $-b^{\left(m\right)}fV$ represents depletion of susceptible target cells due to viral infection, while $\gamma^{\left(m\right)}fV-\delta^{\left(m\right)}V$ captures viral production and clearance, respectively. We adopted this reduced f-V system to preserve mechanistic interpretability while enabling consistent daily coupling with the agent-based transmission model [35], [36]. Details of the posterior-informed weighting scheme used for fitting the within-host ODE parameters, including the inverse-variance construction and robust loss function, are provided in Supplementary Information Section B.

Viral-load–based infectivity is defined through a Hill-type mapping:

(11) $$g_{d}^{\left(m\right)}={\left(\frac{V_{d}^{\left(m\right)}}{V_{\max}^{\left(m\right)}}\right)}^{\alpha },\;\lambda_{d}^{\left(m\right)}=\beta_{\max}^{\left(m\right)}\;g_{d}^{\left(m\right)},\;p_{d}^{\left(m\right)}=1-e^{-\lambda_{d}^{\left(m\right)}},\;\alpha =1.5.$$

The stage-weighted transmission contribution is

(12) $$M^{\left(m\right)}=A_{p}^{\left(m\right)}+p^{\left(m\right)}A_{a}^{\left(m\right)}+\left(1-p^{\left(m\right)}\right)A_{s}^{\left(m\right)}.$$

Calibration ensures matched reproduction numbers:

(13) $$\beta_{\max}^{\left(m\right)}=\frac{R_{0}}{\overset{¯}{k}\;M^{\left(m\right)}}.$$

Daily per-edge infection uses the same union hazard formulation as the baseline ABM, but with time-varying $p_{d}^{\left(m\right)}$ driven by viral load. All variants share identical network structure $\left(n,\overset{¯}{k}\right)$, synergy exponent $\eta_{\mathrm{syn}}$, epidemiological stage structure, and simulation horizon T. Variant dependence in the multi-scale framework enters exclusively through $\left\{b^{\left(m\right)},\gamma^{\left(m\right)},\delta^{\left(m\right)},V_{0}^{\left(m\right)}\right\}$ and the induced transmission scaling parameter $\beta_{\max}^{\left(m\right)}$. Simulation settings: T=150 days, 100 replicates, $R_{0}\in \left\{1.2,1.5,2.0,2.5\right\}$, recording daily trajectories and transmission pairs.

## 3 Results

We assessed how within-host viral kinetics influence population-level SARS-CoV-2 transmission by embedding variant-specific viral trajectories into a multi-scale ABM. Viral-load curves inferred from longitudinal Ct data via hierarchical Bayesian modeling were summarized using a target-cell–limited ODE and translated into time-varying transmission probabilities. A constant-infectiousness baseline model was calibrated to the same basic reproduction number ($R_{0}$) and run on the same contact network to isolate the effect of temporal infectiousness profiles. The piecewise Ct formulation was used exclusively to construct posterior-informed viral load trajectories for ODE parameter estimation and did not directly enter the transmission simulations. Fast-ascent, short-duration kinetics (Omicron) generated earlier and sharper peaks, whereas slower, prolonged kinetics (Alpha) produced delayed and larger outbreaks. These effects arose from the temporal allocation of infectiousness, not from changes in total transmissibility. Overall, within-host viral dynamics emerged as a primary driver of epidemic tempo and peak burden, highlighting their importance for realistic transmission modeling and policy planning.

### 3.1 Posterior estimation of viral load trajectory parameters

We estimated variant-specific within-host viral kinetics using a two-stage procedure combining hierarchical Bayesian inference of Ct time series with mechanistic curve fitting. Ct values, which inversely reflect viral load, were modeled with partial pooling across individuals within each variant to capture shared kinetic structure while allowing individual variation.

Ct trajectories were represented by a piecewise linear function describing proliferation and clearance:

(14) $$Ct\left(t\right)=\left\{\begin{array}{cc} LOD-d_{p}^{\left(m\right)}\left(1+\frac{t-t_{p}^{\left(m\right)}}{w_{p}^{\left(m\right)}}\right),\; & t<t_{p}^{\left(m\right)}\\ LOD-d_{p}^{\left(m\right)}\left(1-\frac{t-t_{p}^{\left(m\right)}}{w_{r}^{\left(m\right)}}\right),\; & t\geq t_{p}^{\left(m\right)} \end{array}\right)$$

where $t_{p}^{\left(m\right)}$ is the Ct nadir (peak viral load), $d_{p}^{\left(m\right)}$ the Ct decline from the detection limit, and $w_{p}^{\left(m\right)},w_{r}^{\left(m\right)}$ the proliferation and clearance durations. This continuous biphasic form provides an interpretable approximation to the natural infection course while avoiding over-parameterization.

Each observation $y_{i}$ was assumed to follow a normal likelihood centered on the latent mean $\mu_{i}^{\left(m\right)}$:

(15) $$y_{i}∼N\left(\mu_{i}^{\left(m\right)},\sigma^{2}\right),$$

with weakly informative priors designed to balance flexibility and physiological plausibility:

(16) $$t_{p}^{\left(m\right)}∼N\left(5,3^{2}\right),\;d_{p}^{\left(m\right)},w_{p}^{\left(m\right)},w_{r}^{\left(m\right)}∼\mathrm{HalfNormal}\left(5\right),\;\sigma ∼\mathrm{HalfNormal}\left(2\right).$$

Left-censored observations contribute via $P\left(y_{i}\leq \mathrm{LOD}|\mu_{i}^{\left(m\right)},\sigma \right)=\Phi \;\left(\left(\mathrm{LOD}-\mu_{i}^{\left(m\right)}\right)/\sigma \right)$, ensuring the likelihood accounts for measurements at or beyond the detection limit. To stabilize Hamiltonian Monte Carlo, a non-centered parameterization was used for episode-level effects, and all duration parameters were constrained to be positive.

To harmonize Ct values from different assays, we converted them into $\log_{10}$ viral load using the calibration relationship

(17) $$\log_{10}V=\frac{Ct-b}{m_{c}}+\log_{10}A,$$

where $m_{c}$ and b denote the slope and intercept of the Ct–$\log_{10}V$ regression, and A is a scaling constant converting to genome equivalents per milliliter (GE/mL). For all analyses, we used $m_{c}=-3.61$, b=40.94, and A=250, following empirically validated calibration standards [22]. Posterior sampling was performed via Hamiltonian Monte Carlo. The resulting posterior estimates for each variant are summarized in Table 2. For interpretability, we report posterior means and standard deviations; alternative summaries (medians and 95% credible intervals) are provided in Table 2. In addition, the resulting posterior distributions can be found in Figure B.2.

Posterior estimates revealed clear variant-specific differences in within-host viral kinetics. **Omicron** exhibited the fastest dynamics, reaching its Ct nadir at approximately 2.3 days with rapid clearance ($w_{r}^{\left(m\right)}\approx 8.4$ days), indicating swift replication and turnover. **Delta** showed a moderate replication pace ($t_{p}^{\left(m\right)}\approx 4.8$ days) and slower clearance ($w_{r}^{\left(m\right)}\approx 10.6$ days), suggesting prolonged viral persistence. In contrast, **Alpha** displayed the slowest and most extended infection course ($t_{p}^{\left(m\right)}\approx 6.5$ days, $w_{r}^{\left(m\right)}\approx 14$ days), consistent with gradual proliferation and delayed recovery. These posterior differences supply the variant-specific temporal heterogeneity that is subsequently propagated through the network in the multi-scale transmission model. Importantly, the inferred temporal patterns are consistent with previous model-based reconstructions of SARS-CoV-2 viral load dynamics obtained through alternative mathematical frameworks [35]. The qualitative agreement across independent modeling approaches suggests that the estimated viral trajectories capture robust features of SARS-CoV-2 infection kinetics rather than artifacts of a specific inference method.

To mechanistically interpret these trajectories, we fitted a target-cell–limited ODE model to the posterior mean viral load curves ($\log_{10}V\left(t\right)$) using robust, weighted nonlinear least squares. The model describes the coupled dynamics between the susceptible cell fraction f(t) and free virus V(t):

(18) $$\frac{df}{dt}=-b^{\left(m\right)}fV,$$

(19) $$\frac{dV}{dt}=\gamma^{\left(m\right)}fV-\delta^{\left(m\right)}V,$$

where $b^{\left(m\right)}$ is the infection rate constant, $\gamma^{\left(m\right)}$ the rate of viral production, $\delta^{\left(m\right)}$ the clearance rate, and $V_{0}^{\left(m\right)}$ the estimated initial viral load. We fixed f(0)=1 and $V\left(0\right)=V_{0}^{\left(m\right)}$.

**Table 2:** Comparison of inferred viral kinetics parameters for SARS-CoV-2 variants.

(a) Posterior summaries of variant-specific Ct trajectory parameters. Each entry is mean ± SD.$t_{p}^{\left(m\right)}$: time of Ct nadir (day); $d_{p}^{\left(m\right)}$: Ct decline from detection limit (Ct cycles); $w_{p}^{\left(m\right)}$: proliferationduration (day); $w_{r}^{\left(m\right)}$: clearance duration (day).				

Variant	$t_{p^{\left(m\right)}}$ (day)	$d_{p^{\left(m\right)}}$ (Ct)	$w_{p^{\left(m\right)}}$ (day)	$w_{r^{\left(m\right)}}$ (day)

Alpha	6.47 ± 1.10	13.83 ± 1.81	9.50 ± 1.87	14.01 ± 1.59
Delta	4.83 ± 1.75	5.55 ± 2.59	7.64 ± 2.69	10.60 ± 3.38
Omicron	2.33 ± 0.85	11.29 ± 1.79	8.27 ± 2.05	8.38 ± 2.09

(b) Estimated within-host ODE parameters from robust, weighted least-squares fits. Each entry ismean ± SE. Units: $b^{\left(m\right)}$ (mL⋅GE^−1^⋅day^−1^), $\gamma^{\left(m\right)}$ (day^−1^), $\delta^{\left(m\right)}$ (day^−1^), $V_{0}^{\left(m\right)}$ (GE/mL).				

Variant	$b^{\left(m\right)}$ (mL ⋅ GE^−1^⋅ day^−1^)	$\gamma^{\left(m\right)}$ (day^−1^)	$\delta^{\left(m\right)}$ (day^−1^)	$V_{0^{\left(m\right)}}$ (GE/mL)

Alpha	(1.78 ± 0.10)×$10^{-7}$	2.53 ± 0.07	1.52 ± 0.07	46.83 ± 2.23
Delta	(1.57 ± 0.17)×$10^{-5}$	1.46 ± 0.08	0.88 ± 0.09	123.90 ± 5.48
Omicron	(2.41 ± 0.68)$\times 10^{-7}$	9.05 ± 2.26	8.02 ± 2.28	628.01 ± 44.02

Weighted residuals were defined by

(20) $$w_{j}^{\left(m\right)}=\frac{1}{s_{j}^{2}+ɛ},\;ɛ=10^{-8},$$

where $s_{j}$ represents the posterior uncertainty at each time point, reducing the influence of less certain estimates. Parameters $\theta^{\left(m\right)}=\left(\log b^{\left(m\right)},\log\gamma^{\left(m\right)},\log\delta^{\left(m\right)},\log V_{0}^{\left(m\right)}\right)$ were estimated by minimizing a robust soft-$L_{1}$ objective:

(21) $$\underset{\theta^{\left(m\right)}>0}{\min}\underset{j}{\sum }\rho \left(\sqrt{w_{j}^{\left(m\right)}}\;\left[\mu \left(t_{j};\theta^{\left(m\right)}\right)-{\overset{¯}{y}}_{j}\right]\right).$$

Ten random multistarts were used to enhance global convergence, and parameter uncertainty was approximated from the inverse Hessian via the delta method. The estimated ODE parameters for each variant are listed in Table 2. We emphasize that the ODE fit is used as a mechanistic summary of the posterior trajectories—not as a second independent inference—so that replication ($\gamma^{\left(m\right)}$), clearance ($\delta^{\left(m\right)}$), and the infection rate constant ($b^{\left(m\right)}$) provide interpretable links to the transmission module.

The mechanistic fits reproduced the Ct-derived $\log_{10}V$ curves with high fidelity. Delta exhibited the highest infection rate constant ($b^{\left(m\right)}$), indicating efficient within-host amplification. Omicron had the largest clearance rate ($\delta^{\left(m\right)}$) and initial viral load ($V_{0}^{\left(m\right)}$), reflecting rapid replication and fast turnover. Alpha displayed slower infection and clearance kinetics, consistent with its extended infection period. Fig. 2 visualizes the full fitted results: Panel A reports estimated within-host parameters by variant, Panel B shows piecewise-linear Ct fits with 95% posterior intervals, and Panel C displays the corresponding ODE-simulated $\log_{10}V\left(t\right)$. The close correspondence between Panels B and C confirms internal consistency between empirical trajectory fitting and mechanistic simulation.

**Fig. 3:**
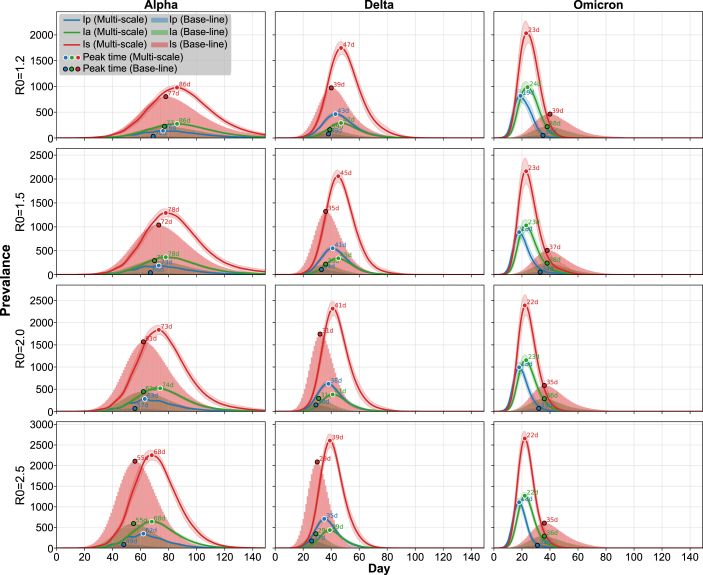
Comparative epidemic dynamics across SARS-CoV-2 variants. Each row corresponds to a basic reproduction number $R_{0}\in \left\{1.2,1.5,2.0,2.5\right\}$, and each column presents epidemic trajectories for the Alpha, Delta, and Omicron variants. For each panel, trajectories from the multi-scale and base-line models are visualized together. The multi-scale model is shown as a solid curve (mean) with a shaded 95% confidence interval, while the base-line model is represented as a bar-style shaded envelope centered on its mean. Peaks for each model are indicated by circular markers, enabling direct comparison under matched $R_{0}$ and variant conditions.

### 3.2 Model validation and comparison of multi-scale and base-line frameworks

To assess how variant-specific within-host kinetics influence epidemic dynamics, we compared a multi-scale model incorporating viral load–dependent infectiousness with a baseline model assuming constant transmissibility. Both models were calibrated to the same basic reproduction number ($R_{0}$), ensuring that observed differences arise from temporal infectiousness patterns rather than average transmission potential. Variant-specific kinetic parameters (b, γ, δ; Table 2) determine replication and clearance dynamics and therefore govern transmission timing in the multi-scale framework. We evaluated epidemic outcomes in terms of tempo (peak timing), magnitude (peak size and final epidemic size), and variability (95% confidence intervals). We additionally report a Peak-Advance Index (PAI),

(22) $$\mathrm{PAI}={\mathrm{PeakDay}}_{\text{MS}}-{\mathrm{PeakDay}}_{\text{BL}},$$

where PAI<0 indicates an earlier peak under the multi-scale model.

Simulations were performed on a scale-free network of 10,000 individuals, with each scenario repeated 100 times to capture stochastic variability. Fig. 3 shows epidemic trajectories for each variant and $R_{0}$ value under the multi-scale and baseline models. We tracked pre-symptomatic ($I_{p}$), asymptomatic ($I_{a}$), and symptomatic ($I_{s}$) infections to characterize the time distribution of infectious individuals.

**Fig. 4:**
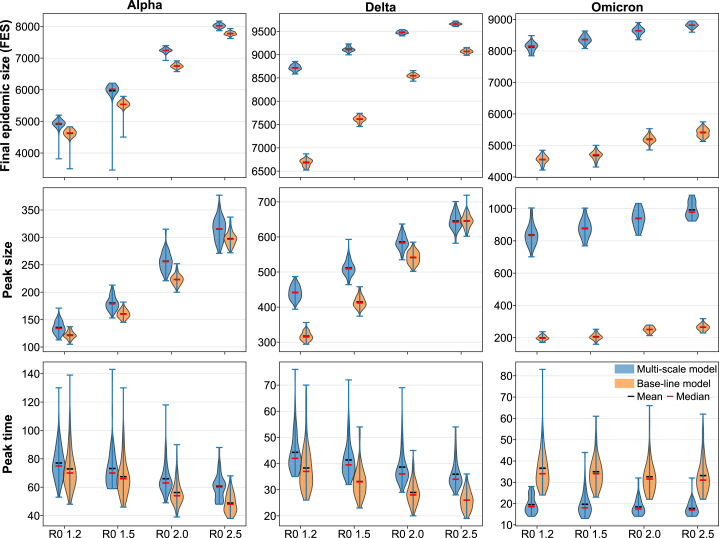
Epidemic Outcomes Across Variants Under Multi-Scale and Base-line Models. Each column corresponds to one of the SARS-CoV-2 variants—Alpha, Delta, and Omicron—while each row represents key epidemic indicators: final epidemic size (FES), peak size, and peak timing. Results are shown for different basic reproduction numbers ($R_{0}$) ranging from 1.2 to 2.5. Each violin plot summarizes 100 stochastic simulation replicates, with white bars indicating the mean values.

Across nearly all variants and $R_{0}$ values, the multi-scale model produced higher or comparable peaks relative to the baseline model, and the magnitude of this difference generally increased with $R_{0}$. Importantly, peak timing diverged: Alpha and Delta peaked later under the multi-scale model, whereas Omicron peaked earlier. Estimated shifts ranged from ＋4 to ＋12 days for Alpha, ＋6 to ＋10 days for Delta, and −14 to −17 days for Omicron across $R_{0}$ values, closely matching the ordering of viral proliferation rates (Alpha < Delta < Omicron; Fig. 2C). These results show that faster within-host viral expansion translates into accelerated epidemic spread at the population level, even under equal $R_{0}$ calibration.

Fig. 4 shows that viral kinetic differences generate distinct epidemic outcomes. Peak magnitudes differed substantially across variants, with Omicron exhibiting the largest peaks under the multi-scale framework, particularly at higher $R_{0}$ values. Although peak size scaled with $R_{0}$, the qualitative dependence on variant-specific kinetics remained consistent across transmission intensities, indicating biologically driven effects rather than stochastic variation. These results reflect systematic differences in viral kinetics. Alpha’s slow rise and sustained high viral load prolonged infectiousness, yielding delayed and moderately amplified epidemic waves. Delta exhibited faster proliferation and clearance, producing moderately earlier peaks with reduced amplification relative to the baseline model. Omicron’s rapid rise and clearance concentrated infectiousness into a shorter window, leading to earlier and sharper peaks in the multi-scale model – a reversal of the Alpha and Delta patterns – demonstrating that accelerated within-host kinetics compress transmission timing and cannot be replicated by constant-rate models.

Overall, the timing of infectiousness, not only its total magnitude, governs epidemic tempo and severity. Faster proliferation accelerates and sharpens outbreaks, whereas slower dynamics delay and amplify peaks, even under identical $R_{0}$. Additional quantitative comparisons supporting these trends are provided in Supplementary Figure B.3 and Supplementary Table B.1. Under the multi-scale model, Omicron is associated with the largest relative increases in peak size and final epidemic size (FES), whereas Alpha and Delta exhibit comparatively smaller changes. Furthermore, Omicron shows the greatest advancement in peak timing across the scenarios considered.

**Fig. 5:**
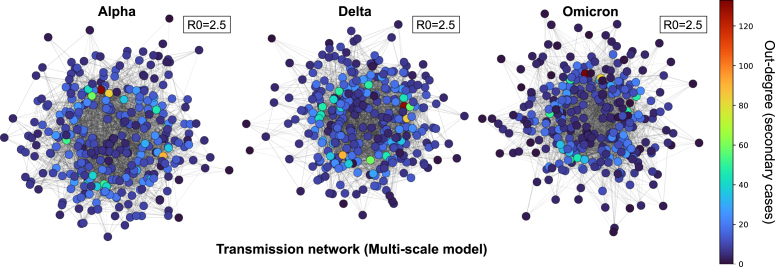
Transmission networks based on transmission pairs traced from infected individuals. This figure visualizes representative portions of transmission networks reconstructed by tracing transmission pairs in each scenario. Results shown correspond to simulations with $R_{0}=2.5$, and columns represent the Alpha, Delta, and Omicron variants. Nodes are colored according to the number of secondary infections they generated; redder nodes indicate individuals with greater transmission impact, highlighting key contributors to secondary spread. The full transmission networks for all $R_{0}$ values and variants under both the multi-scale and base-line models can be found in Figure C.2, C.3.

**Fig. 6:**
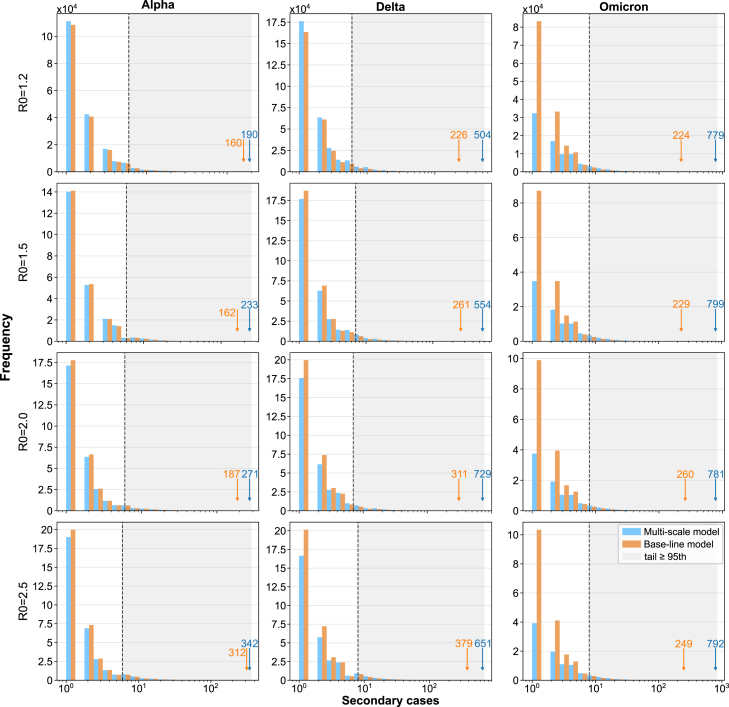
Frequency distribution of secondary cases by variant and $R_{0}$. This figure presents the distribution of secondary infections generated across scenarios. Columns correspond to SARS-CoV-2 variants, and rows represent basic reproduction numbers $R_{0}$. Within each panel, results from the multi-scale and base-line models are shown together to facilitate direct comparison. The individual responsible for the largest number of secondary infections in each scenario is highlighted, with both the position and the number of secondary infections explicitly indicated.

The multi-scale model also generated realistic transmission heterogeneity without imposing overdispersion parameters. Reconstructed transmission networks (Fig. 5) showed a minority of individuals accounting for disproportionately many infections. We further characterized this behavior through secondary-infection distributions (Fig. 6). Both the multi-scale and baseline models exhibited long-tailed offspring distributions, indicating rare but impactful events. However, the multi-scale model generally produced heavier tails and higher maximum secondary case counts, whereas the baseline model concentrated transmission among individuals causing only a few infections. Thus, incorporating time-varying viral kinetics increases the probability of extreme transmission events and strengthens the upper tail of the secondary infection distribution relative to constant-infectivity assumptions. To quantify tail behavior, we use the complementary cumulative distribution function (CCDF), defined for a random variable X as CCDF(x)=Pr(X≥x)=1−CDF(x). CCDFs (Figure C.1) approached the canonical $x^{-2}$ decay, consistent with heavy-tailed epidemic processes.

Variant-specific viral kinetics reshaped epidemic dynamics under identical $R_{0}$, affecting peak timing, magnitude, and the probability of superspreading. Fast replication compressed infectiousness, accelerating peaks (Omicron), while slower clearance prolonged waves and increased epidemic size (Alpha). The multi-scale model reproduced realistic transmission heterogeneity and more pronounced superspreading patterns than those observed under constant-infectivity models, underscoring the importance of biologically grounded β(V) functions for evaluating variant-specific epidemic potential and tail risk under fixed $R_{0}$.

## 4 Discussion

We developed a mechanistic multi-scale agent-based model that couples within-host viral kinetics with population-level transmission to explain how biological variation across SARS-CoV-2 variants shapes epidemic behavior. Grounded in a One Health perspective that links human biology, contact behavior, and environmental context, this framework advances data-integrated epidemic estimation and risk-assessment tools. By incorporating empirically inferred viral-load trajectories and calibrating both the multi-scale and baseline models to a common basic reproduction number ($R_{0}$), we isolate temporal heterogeneity in infectiousness as a primary driver of epidemic tempo, magnitude, and duration. Differences in epidemic shape therefore arise not from changes in mean transmissibility, but from variant-specific replication and clearance profiles. Consistent with previous work, the qualitative shape of the inferred viral-load trajectories is consistent with results obtained using alternative modeling approaches that couple within-host viral kinetics with population-level transmission dynamics [35]. This agreement across independent frameworks suggests that the temporal structure of infectiousness identified here reflects robust biological characteristics of SARS-CoV-2 infection rather than artifacts of a specific modeling formulation.

Time-varying transmission probability captures dynamic shifts in infection burden as dominant variants change. Fast-ascending variants such as Omicron generate markedly earlier and sharper peaks, whereas slower dynamics in Alpha delay epidemic waves; Delta exhibits intermediate timing shifts but less dramatic amplification than Omicron. These emergent patterns, difficult to reproduce under constant-transmissibility assumptions, highlight the importance of capturing biological time structure for One Health-aligned epidemic assessment. Bayesian inference of longitudinal Ct trajectories revealed clear kinetic contrasts across variants – rapid rise and clearance for Omicron, prolonged high viral load for Alpha, intermediate kinetics for Delta – consistent with empirical virology studies [22], [37], [38]. When mapped to transmission hazards, these differences yielded markedly earlier peaks and substantial amplification in epidemic magnitude for Omicron, whereas Alpha exhibited delayed epidemic waves with more moderate increases in final size, aligning with theoretical links between infection-age infectiousness and epidemic growth [7], [8].

A key strength of the agent-based implementation is its ability to generate heterogeneous transmission and superspreading events without ad-hoc assumptions. Within-host heterogeneity is often incorporated into compartmental models through age-of-infection structures or multiple infectious cohorts, which expand the population-level state space while preserving analytical tractability [35], [36]. Complementary macroscopic approaches also account for contact heterogeneity via degree-structured, pair-approximation, or kinetic formulations [39], [40]. In contrast, we directly couple the within-host viral-kinetics model to an individual-based transmission process on an explicit contact network, enabling time-varying infectiousness to interact with network heterogeneity and stochastic contacts under controlled population-level conditions. While the SEIpIaIsR structure defines infection states and transition processes, spatial and contact heterogeneity do not arise from these compartmental equations themselves. Instead, heterogeneity emerges from the explicit network-based agent implementation, where degree variability, clustering, and stochastic contact processes shape transmission pathways. In particular, all simulations were conducted on a scale-free contact network characterized by heavy-tailed degree distributions, a structure known to intrinsically support transmission heterogeneity and rare high-impact events. Importantly, time-varying infectiousness does not create superspreading events de novo, but instead increases the probability and magnitude of extreme transmission outcomes already permitted by network structure and stochastic contact processes [9], [41], [42]. Individual viral trajectories interacting with network structure produced realistic overdispersion and rare high-impact events, reinforcing the relevance of this approach for One Health-oriented risk assessment where context and stochasticity matter.

Across $R_{0}$ values and metrics – final size, peak height, peak timing – the multi-scale and baseline models diverged across most scenarios, confirming that time-varying infectiousness is not a secondary refinement but a fundamental determinant of epidemic dynamics. Integrating biological kinetics into transmission models enhances realism and early-warning capacity, complementing One Health priorities focused on data integration, mechanistic inference, and proactive preparedness.

Methodologically, this study advances hierarchical Bayesian modeling of censored longitudinal Ct data and links those estimates to a network-based epidemic model. Censoring-aware inference and assay standardization ensure physiological interpretability [43], [44], [45], enabling a coherent bridge from viral biology to epidemic outcomes [12], [14], [46], [47]. Limitations include computational demands, variation in viral-load sampling, and the need to explicitly model immunity, vaccination, and reinfection in later pandemic phases [38], [48].

In summary, modest differences in viral replication and clearance propagate to substantial differences in epidemic timing, peak burden, and final epidemic size. Embedding variant-specific viral kinetics in a calibrated ABM establishes a coherent biological-to-population causal chain, strengthens One Health-aligned data integration, and improves timing-sensitive intervention planning. This framework provides a foundation for risk assessment and preparedness against future respiratory pathogens with dynamic infectiousness profiles.

## CRediT authorship contribution statement

**Hyosun Lee:** Writing – original draft, Visualization, Software, Methodology, Investigation, Funding acquisition, Formal analysis, Data curation. **Byul Nim Kim:** Writing – original draft, Validation, Supervision, Project administration, Methodology, Investigation, Formal analysis. **Sunmi Lee:** Writing – original draft, Validation, Supervision, Resources, Project administration, Investigation, Funding acquisition, Formal analysis, Conceptualization.

## Funding

This work was supported by the 10.13039/501100003725National Research Foundation of Korea (NRF), South Korea grant funded by the Korea government (MSIT) (No. RS-2022-NR070839, RS-2024-00351984). This work also received support from the BK21 FOUR program of the Graduate School, Kyung Hee University (GS-1-JO-NON-20240367). Funding was additionally provided by Basic Science Research Program through the National Research Foundation of Korea (NRF) funded by the Ministry of Education (No. RS-2025-25429748).

## Declaration of competing interest

The authors declare that they have no known competing financial interests or personal relationships that could have appeared to influence the work reported in this paper.

## Data Availability

The data used in this study were obtained from the Korea Disease Control and Prevention Agency (KDCA) [2]. In addition, publicly available SARS-CoV-2 Ct value data from [22] were utilized.

## Appendix A Supplementary data

MMC S1 Supplementary materials with detailed model descriptions, viral kinetics inference, validation, and network structure. MMC S1
