# Supplementary material for: Integrating viral kinetics and population spread in a one health framework to explain variant-specific epidemic dynamics
Source: One Health. 2026 Mar 17;22:101389. doi: 10.1016/j.onehlt.2026.101389 (PMC13010435; doi:10.1016/j.onehlt.2026.101389)
Supplement: MMC S1 — Supplementary materials with detailed model descriptions, viral kinetics inference, validation, and network structure. [file mmc1.pdf]

Supplementary Information

A. Variant-Specific Viral Kinetics and Multi-Scale Transmission Modeling Framework

To characterize individual-level viral kinetics, we used publicly available longitudinal cycle-threshold (Ct) measurements from RT-qPCR assays [1]. Each infection episode includes repeated Ct values with metadata describing variant, sampling time relative to detection, vaccination status, antibody titer category, and symptom presentation. Within each episode, observations were time-ordered to reconstruct viral trajectories capturing both proliferation and clearance phases. Episodes with insufficient sampling density or negligible Ct variation were excluded to ensure reliable parameter inference.

Because Ct values are inversely related to viral load and vary by assay platform, we applied the inter-assay linear calibration from [1]:

$$Ct' = a_0 + a_1\, Ct, \qquad (a_0, a_1) = (-6.25,\ 1.34). \tag{1}$$

To harmonize detection limits, calibrated values were truncated at the assay-specific limit of detection (LOD) and treated as left-censored in subsequent inference:

$$Ct' \ \leftarrow\ \min\{Ct',\ \mathrm{LOD}\}, \qquad y = \min\{Ct',\ \mathrm{LOD}\} \text{ with left-censoring at LOD.} \tag{2}$$

We required trajectories to contain sufficient measurable information:

$$\min(Ct') \le 32 \quad \text{and} \quad \#\{\ Ct' < \mathrm{LOD}\ \} \ge 3, \tag{3}$$

ensuring at least three quantifiable points and a sufficiently low minimum Ct to capture the proliferation phase. Sampling times were expressed in days relative to the first positive test and normalized so that the earliest observation in each episode corresponded to $t = 0$, enabling consistent temporal alignment across individuals and variants.

Host and clinical covariates were standardized prior to inference. Vaccination status was fixed at detection to reflect immune status near infection onset. Antibody titers were collapsed into ordered categories, assigning the highest available value when multiple measurements occurred. Symptom status was reduced to a binary variable (symptomatic vs. asymptomatic) using a precedence rule when discordant records appeared. All preprocessing (time normalization, censoring, and covariate harmonization) was performed separately for each variant, yielding standardized viral-load trajectories suitable for Bayesian inference and mechanistic within-host modeling.

A.1. Base-line ABM without within-host dynamics (network simulation)

The baseline agent-based model simulates SARS-CoV-2 transmission on a static undirected contact network with mean degree $\bar{k}$, using daily time steps for $T = 150$ days. Throughout this Supplementary section, the superscript (m) denotes a variant index, with $m \in \{\text{Alpha}, \text{Delta}, \text{Omicron}\}$. All parameters carrying the superscript (m) are variant-specific, including the branching probability $p^{(m)}_{\text{asym}}$, the within-host kinetic parameters $(b^{(m)}, \gamma^{(m)}, \delta^{(m)}, V^{(m)}_0)$, the stage durations $(T^{(m)}_p, T^{(m)}_a, T^{(m)}_s)$, and the calibrated transmission scales $(\beta^{(m)}_{\max}, \beta^{(m)}_{\ell})$. Parameters without the superscript (m) are shared across variants. Each individual occupies one of six states: susceptible (S), exposed (E), presymptomatic infectious (I_p), asymptomatic infectious (I_a), symptomatic infectious (I_s), or recovered (R). A susceptible individual becomes infected through contact with infectious neighbors and transitions to E. After a latent period, individuals transition from E to I_p. Upon leaving I_p, individuals branch to I_a with probability $p^{(m)}_{\text{asym}}$ or to I_s with probability $1 - p^{(m)}_{\text{asym}}$, and then recover to R. Recovered individuals are removed from transmission.

The presymptomatic state is explicitly retained to represent presymptomatic transmission, to preserve timing effects under fixed $\mathcal{R}_0$, and to enforce an identical stage partition across baseline and multi-scale models so that differences can be attributed to time-varying infectiousness rather than to compartmental definitions.

For a susceptible individual j on day t, let $\mathcal{N}^{\text{inf}}_j(t)$ denote the set of infectious neighbors (those in I_p, I_a, or I_s). Each infectious neighbor contributes a per-edge infection probability that depends only on the neighbor's stage:

$$p_i(t) = \begin{cases} \beta^{(m)}_p, & i \in I_p, \\ \beta^{(m)}_a, & i \in I_a, \\ \beta^{(m)}_s, & i \in I_s, \end{cases} \qquad \beta^{(m)}_a = \eta_a \beta^{(m)}_p,\ \beta^{(m)}_s = \eta_s \beta^{(m)}_p. \tag{4}$$

We fix $\eta_a = 0.2$ and $\eta_s = 0.5$ in all experiments. These fixed scalings are chosen within empirically supported ranges to reflect substantial presymptomatic transmission with attenuated asymptomatic and post-symptom infectiousness, and to improve identifiability and cross-variant comparability by avoiding over-parameterization of stage-specific transmissibility.

Assuming conditional independence of infection events across edges at the daily scale, the probability that j becomes infected on day t is

$$p_{\text{union}}(j,t) = 1 - \prod_{i \in \mathcal{N}_j^{\text{inf}}(t)} \big(1 - p_i(t)\big). \tag{5}$$

To allow non-additive interaction among multiple exposures within a day, we apply a synergy mapping

$$p_{\text{tot}}(j,t) = 1 - \big(1 - p_{\text{union}}(j,t)\big)^{\eta_{\text{syn}}}, \tag{6}$$

where $\eta_{\text{syn}} > 0$ controls sub- (< 1) versus super-additive (> 1) aggregation, and $\eta_{\text{syn}} = 1$ recovers the independence model. When infection occurs, the infector is sampled from $\mathcal{N}_j^{\text{inf}}(t)$ with probability proportional to the stage-specific per-edge probabilities, ensuring consistent attribution. We emphasize that η_{syn} is introduced only in the baseline ABM as an optional, phenomenological adjustment to compensate for the structural rigidity of time-invariant per-stage transmissibility; in the multi-scale ABM, analogous nonlinearities arise endogenously through viral-load–modulated hazards and network-mediated multi-neighbor exposure, so an additional synergy exponent is not required.

A.2. Compartmental SEIpIaIsR representation of the baseline ABM (interpretation)

To facilitate interpretation and to connect the ABM to standard compartmental notation, we report a discrete-time mean-field SEIpIaIsR approximation of the baseline process. This representation is not used to generate results; all reported outcomes are produced by the individual-based network simulation.

Let $S(t), E(t), I_p(t), I_a(t), I_s(t), R(t)$ denote population counts and let n be population size. Under a sparse-network mean-field approximation, the probability that a susceptible avoids infection across $\bar{k}$ contacts is approximated by a product of "no infection" terms contributed by each infectious stage. Incorporating the synergy exponent yields:

$$\begin{aligned}
\Delta S(t) &= -S(t) \left\{ 1 - \left[(1-\beta_p^{(m)})^{\frac{\bar{k}\, I_p(t)}{n}} (1-\beta_a^{(m)})^{\frac{\bar{k}\, I_a(t)}{n}} (1-\beta_s^{(m)})^{\frac{\bar{k}\, I_s(t)}{n}} \right]^{\eta_{\text{syn}}} \right\}, \\
\Delta E(t) &= \ S(t) \left\{ 1 - \left[(1-\beta_p^{(m)})^{\frac{\bar{k}\, I_p(t)}{n}} (1-\beta_a^{(m)})^{\frac{\bar{k}\, I_a(t)}{n}} (1-\beta_s^{(m)})^{\frac{\bar{k}\, I_s(t)}{n}} \right]^{\eta_{\text{syn}}} \right\} - \sigma^{(m)} E(t), \\
\Delta I_p(t) &= \sigma^{(m)} E(t) - \kappa^{(m)} I_p(t), \\
\Delta I_a(t) &= p_{\text{asym}}^{(m)} \kappa^{(m)} I_p(t) - \gamma_a^{(m)} I_a(t), \\
\Delta I_s(t) &= (1 - p_{\text{asym}}^{(m)}) \kappa^{(m)} I_p(t) - \gamma_s^{(m)} I_s(t), \\
\Delta R(t) &= \gamma_a^{(m)} I_a(t) + \gamma_s^{(m)} I_s(t),
\end{aligned} \tag{7}$$

where $\sigma^{(m)}, \kappa^{(m)}, \gamma_a^{(m)}, \gamma_s^{(m)}$ are daily transition rates and $T_E^{(m)} = 1/\sigma^{(m)}$, $T_p^{(m)} = 1/\kappa^{(m)}$, $T_a^{(m)} = 1/\gamma_a^{(m)}$, $T_s^{(m)} = 1/\gamma_s^{(m)}$ are the corresponding mean stage durations.

The synergy exponent η_{syn} appears as an exponent on the "no-infection" product term. This mirrors the individual-level transformation $p_{\text{tot}} = 1 - (1 - p_{\text{union}})^{\eta_{\text{syn}}}$, i.e., synergy modifies how multiple simultaneous exposures are aggregated at the day scale.

A.3. Multi-scale ABM with within-host dynamics (ODE-coupled transmission)

The multi-scale model couples within-host viral kinetics and between-host transmission on the same contact network. The between-host state space is identical to the baseline model (S, E, I_p, I_a, I_s, R) with the same branching probability $p_{\text{asym}}^{(m)}$ and the same relative infectiousness scaling $\eta_a = 0.2$, $\eta_s = 0.5$. The difference is that per-edge transmission varies over infection age and is generated from viral load dynamics rather than being constant within stages.

Within-host dynamics follow a target-cell–limited model:

$$\frac{df}{dt} = -b^{(m)} f V, \qquad \frac{dV}{dt} = \gamma^{(m)} f V - \delta^{(m)} V, \qquad (f, V)\Big|_{t=0} = (1, V_0^{(m)}). \tag{8}$$

Variant-specificity enters through $(b^{(m)}, \gamma^{(m)}, \delta^{(m)}, V_0^{(m)})$. Individual heterogeneity is represented by sampling these parameters around variant-specific means with variant-specific standard deviations (Table A.2), and each infected individual retains its sampled parameter set throughout a run.

The within-host ODE is integrated over each day, and end-of-day values define the next day's initial condition. Infectivity for individual i on day d is derived via a Hill mapping:

$$g_{i,d} = \left(\frac{V_{i,d}}{V_{\max}}\right)^{\alpha}, \qquad \alpha = 1.5, \tag{9}$$

where $V_{\max}$ is a fixed normalizer. $V_{\max}$ is computed as the maximum viral load along a sufficiently long reference trajectory generated under the variant mean parameters, with an additional buffer beyond the nominal infectious window to cover tail durations. This prevents spurious cross-variant rescaling induced by insufficient time horizons.

Daily per-edge hazards and probabilities are defined as

$$\lambda_{i,d} = \beta_{\max}^{(m)} g_{i,d}, \qquad p_{i,d} = 1 - e^{-\lambda_{i,d}}. \tag{10}$$

Stage-specific relative infectiousness is applied multiplicatively: asymptomatic hazards are scaled by η_a, symptomatic hazards are scaled by η_s, and presymptomatic hazards use $\eta_p = 1$. Multi-neighbor exposure is aggregated by the union-of-independent-hazards rule (without an additional synergy exponent in the multi-scale model), and infectors are attributed proportionally to per-edge probabilities.

For interpretation, a mean-field SEIpIaIsR approximation can be written by replacing constant per-edge probabilities with time-varying, viral-load–modulated stage averages:

$$\begin{aligned}
\Delta S(t) &= -S(t)\left\{1 - \left[(1-\bar{p}_p(t))^{\frac{\bar{k}\, I_p(t)}{n}} (1-\bar{p}_a(t))^{\frac{\bar{k}\, I_a(t)}{n}} (1-\bar{p}_s(t))^{\frac{\bar{k}\, I_s(t)}{n}}\right]\right\},\\
\Delta E(t) &= S(t)\left\{1 - \left[(1-\bar{p}_p(t))^{\frac{\bar{k}\, I_p(t)}{n}} (1-\bar{p}_a(t))^{\frac{\bar{k}\, I_a(t)}{n}} (1-\bar{p}_s(t))^{\frac{\bar{k}\, I_s(t)}{n}}\right]\right\} - \sigma^{(m)} E(t),\\
\Delta I_p(t) &= \sigma^{(m)} E(t) - \kappa^{(m)} I_p(t),\\
\Delta I_a(t) &= p_{\text{asym}}^{(m)} \kappa^{(m)} I_p(t) - \gamma_a^{(m)} I_a(t),\\
\Delta I_s(t) &= (1 - p_{\text{asym}}^{(m)}) \kappa^{(m)} I_p(t) - \gamma_s^{(m)} I_s(t),\\
\Delta R(t) &= \gamma_a^{(m)} I_a(t) + \gamma_s^{(m)} I_s(t),
\end{aligned} \tag{11}$$

where

$$\bar{p}_p(t) = 1 - \exp\!\big(-\beta_{\max}^{(m)}\, \bar{g}_p(t)\big), \quad \bar{p}_a(t) = 1 - \exp\!\big(-\eta_a\, \beta_{\max}^{(m)}\, \bar{g}_a(t)\big), \quad \bar{p}_s(t) = 1 - \exp\!\big(-\eta_s\, \beta_{\max}^{(m)}\, \bar{g}_s(t)\big), \tag{12}$$

with the stage-specific mean infectivity

$$\bar{g}_\ell(t) = \frac{1}{N_\ell(t)} \sum_{i \in I_\ell(t)} \left(\frac{V_i(t)}{V_{\max}}\right)^{\alpha}, \qquad \ell \in \{p, a, s\}. \tag{13}$$

Here $N_\ell(t) = |I_\ell(t)|$. When $N_\ell(t) = 0$, we set $\bar{g}_\ell(t) = 0$ and thus $\bar{p}_\ell(t) = 0$. This representation corresponds to $\eta_{\text{syn}} = 1$ and is used solely for interpretation; simulation outputs are generated by the individual-level network model.

A.4. Variant-specific parameterization and $\mathcal{R}_0$ calibration (detailed)

Executive summary. (i) In the multi-scale ABM, we calibrate $\beta_{\max}^{(m)}$ so that the expected number of secondary infections equals the target $\mathcal{R}_0$ under the shared network degree $\bar{k}$ by computing a variant-specific "transmission mass" $M^{(m)}$ from the viral-load–derived infectivity profile and stage-timing distributions. (ii) In the baseline ABM,

we determine constant stage-specific hazards so that both $\mathcal{R}_0$ and the expected stage-wise allocation of transmission match the multi-scale model. (iii) Optionally, we tune the baseline synergy exponent η_{syn} to match the multi-scale mean final epidemic size, providing an additional alignment layer when time-invariant stage hazards cannot fully reproduce the outcomes induced by time-varying infectiousness.

We design the simulation study to cleanly separate shared population-level components from variant-specific biological components. The contact network (and $\bar{k}$), the between-host state space S, E, I_p, I_a, I_s, R, and the relative infectiousness scalings $\eta_a = 0.2$ and $\eta_s = 0.5$ are shared across all variants and across both modeling frameworks. Variant differences enter through (i) stage-timing distributions and the branching probability $p_{\text{asym}}^{(m)}$ (Table A.1), and (ii) within-host kinetic parameters $(b^{(m)}, \gamma^{(m)}, \delta^{(m)}, V_0^{(m)})$ (Table A.2). Although the numerical values reported in these supplementary tables are identical to those presented in Table 1 of the main text, they are expressed in a different form to explicitly represent the parameters of the underlying gamma distributions used in the simulations.

Because the ABM evolves in daily time steps, all residence times used in simulation are integer-valued days. We therefore (i) specify continuous-time Gamma distributions using mean–SD moment matching, and (ii) convert continuous draws into discrete days using a mean-preserving discretization so that the discrete-time simulation remains unbiased with respect to the intended continuous-time mean durations.

For a Gamma distribution Gamma(a, θ) with shape a and scale θ, we match a desired mean μ and standard deviation σ using

$$a = \left(\frac{\mu}{\sigma}\right)^2, \qquad \theta = \frac{\sigma^2}{\mu}. \tag{14}$$

To generate an integer day count $D \in \{1, 2, \dots\}$ from a continuous draw $L \geq 0$, we use mean-preserving discretization: write $L = n + u$ where $n = \lfloor L \rfloor$ and $u \in [0, 1)$, then set $D = n + 1$ with probability u and $D = n$ otherwise, enforcing $D \geq 1$. This construction satisfies $\mathbb{E}[D] = \mathbb{E}[L]$ up to truncation at 1 day and avoids the upward bias induced by deterministic ceiling.

We next describe $\mathcal{R}_0$ calibration in the multi-scale ABM and the baseline ABM in a step-by-step manner. Throughout, $\bar{k}$ denotes the mean degree of the shared contact network.

A. $\mathcal{R}_0$ calibration in the multi-scale ABM (within-host driven, time-varying per-edge hazards). In the multi-scale ABM, infectiousness varies with infection age through viral load. For each infected individual, viral load $V^{(m)}(t)$ is generated by the within-host ODE and synchronized to the daily ABM clock by end-of-day aggregation. Daily infectivity is defined by

$$g_d^{(m)} = \left(\frac{V_d^{(m)}}{V_{\max}}\right)^{\alpha}, \qquad \alpha = 1.5, \tag{15}$$

and the per-edge daily infection hazard and probability at infection-age day d are

$$\lambda_d^{(m)} = \beta_{\max}^{(m)} g_d^{(m)}, \qquad p_d^{(m)} = 1 - \exp(-\lambda_d^{(m)}). \tag{16}$$

The calibration objective is to choose $\beta_{\max}^{(m)}$ so that the expected number of secondary infections generated by a typical infected individual equals the target $\mathcal{R}_0$ under the shared network with mean degree $\bar{k}$. Under a sparse-network approximation, the expected number of secondary infections is approximated by $\bar{k}$ multiplied by the expected cumulative per-edge hazard integrated over the infectious history, with branching after I_p accounted for by $p_{\text{asym}}^{(m)}$. This yields a closed-form scaling in terms of an expected "transmission mass" $M^{(m)}$ defined below.

Step A1 (define a sufficiently long infectivity horizon). Let $D_p^{(m)}$ be the presymptomatic duration (days), and let $D_a^{(m)}$ and $D_s^{(m)}$ be the post-branch durations (days) for asymptomatic and symptomatic pathways, respectively. Because these durations are random and may have non-negligible tails, we evaluate infectivity on a horizon H long enough to cover typical realizations, defined as a conservative upper bound based on the mean durations plus an additional buffer. This avoids truncation of the infectivity profile when estimating cumulative contributions.

Step A2 (compute the daily infectivity profile on the horizon). We compute a reference daily sequence $(g_1^{(m)}, \dots, g_H^{(m)})$ from the within-host ODE under variant mean parameters, and define the cumulative sum

$$G^{(m)}(d) = \sum_{u=1}^{d} g_u^{(m)}, \qquad d = 0, 1, \dots, H, \qquad G^{(m)}(0) = 0. \tag{17}$$

Step A3 (Monte Carlo estimation of stage-wise cumulative contributions). We draw M_{MC} independent samples of $(D_p^{(m)}, D_a^{(m)}, D_s^{(m)})$ (integer days) from the variant-specific timing distributions. For each draw, the stage-wise cumulative contributions are

$$A_p^{(m)} = G^{(m)}(D_p^{(m)}) - G^{(m)}(0), \tag{18}$$

$$A_a^{(m)} = \eta_a \left[G^{(m)}(D_p^{(m)} + D_a^{(m)}) - G^{(m)}(D_p^{(m)}) \right], \tag{19}$$

$$A_s^{(m)} = \left[G^{(m)}(D_p^{(m)} + D_s^{(m)}) - G^{(m)}(D_p^{(m)}) \right], \tag{20}$$

where $\eta_a = 0.2$ is applied within the asymptomatic pathway by definition, and the symptomatic scaling $\eta_s = 0.5$ is applied in Step A4 when combining pathway contributions (equivalently, one may define $\tilde{A}_s^{(m)} = \eta_s A_s^{(m)}$; we keep η_s outside here only to make the subsequent branching-and-scaling structure explicit). We then estimate expectations by averaging:

$$A_p^{(m)} = \frac{1}{M_{\text{MC}}} \sum A_p^{(m)}, \quad A_a^{(m)} = \frac{1}{M_{\text{MC}}} \sum A_a^{(m)}, \quad A_s^{(m)} = \frac{1}{M_{\text{MC}}} \sum A_s^{(m)}. \tag{21}$$

Step A4 (combine stage contributions with branching). Branching occurs after I_p. Therefore, the expected total transmission mass is

$$M^{(m)} = A_p^{(m)} + p_{\text{asym}}^{(m)} A_a^{(m)} + (1 - p_{\text{asym}}^{(m)}) \eta_s A_s^{(m)}, \tag{22}$$

where $\eta_s = 0.5$ is the symptomatic transmissibility factor.

Step A5 (solve for $\beta_{\max}^{(m)}$). We set

$$\beta_{\max}^{(m)} = \frac{\mathcal{R}_0}{\bar{k}\, M^{(m)}}. \tag{23}$$

B. Stage-allocation alignment and $\mathcal{R}_0$ calibration in the baseline ABM (constant per-stage probabilities). The baseline ABM uses constant per-edge probabilities $(\beta_p^{(m)}, \beta_a^{(m)}, \beta_s^{(m)})$ within each infectious stage.

Step B1 (define target stage allocation from the multi-scale model). Define stage weights

$$w^{(m)} = \left(A_p^{(m)},\ p_{\text{asym}}^{(m)} A_a^{(m)},\ (1 - p_{\text{asym}}^{(m)}) \eta_s A_s^{(m)}\right), \qquad r^{(m)} = \frac{w^{(m)}}{\sum_\ell w_\ell^{(m)}}. \tag{24}$$

Step B2 (define duration weights for hazard allocation). Let $T_p^{(m)}, T_a^{(m)}, T_s^{(m)}$ denote the mean discrete-day durations. Define

$$W^{(m)} = \left(T_p^{(m)},\ p_{\text{asym}}^{(m)} T_a^{(m)},\ (1 - p_{\text{asym}}^{(m)}) T_s^{(m)}\right). \tag{25}$$

Step B3 (construct reference hazards that preserve allocation ratios). We set reference hazards

$$\tilde{h}_\ell^{(m)} = \frac{r_\ell^{(m)}}{W_\ell^{(m)}}, \qquad \ell \in \{p, a, s\}, \tag{26}$$

and then uniformly rescale them by a scalar $s > 0$:

$$h_\ell^{(m)}(s) = s\, \tilde{h}_\ell^{(m)}. \tag{27}$$

Per-edge transmission probabilities are then

$$\beta_\ell^{(m)}(s) = 1 - \exp\!\left(-h_\ell^{(m)}(s)\right), \qquad \ell \in \{p, a, s\}. \tag{28}$$

Table A.1
Variant-specific between-host timing and branching parameters (mean μ and SD σ, days). Gamma distributions are moment-matched with shape $a = (\mu/\sigma)^2$ and scale $\theta = \sigma^2/\mu$.

Variant	$L_{E\to I_p}$ (μ, σ)	$L_{I_p\to \text{branch}}$ (μ, σ)	$L_{I_{a/s}\to R}$ (μ, σ)	p_{asym}
Alpha	(4.94, 2.19)	(0.70, 1.50)	(13.30, 1.95)	0.22
Delta	(4.40, 2.46)	(0.64, 1.50)	(6.00, 2.22)	0.142
Omicron	(2.58, 1.52)	(0.83, 1.20)	(6.00, 3.71)	0.324

Table A.2
Variant-specific within-host kinetic parameters (mean ± SD). Individual parameters are sampled around the mean with the listed SD; positivity is enforced by truncation/clipping.

Variant	b (mean ± SD)	γ (mean ± SD)	δ (mean ± SD)	V_0 (mean ± SD)
Alpha	$1.78\times10^{-7} \pm 1.0\times10^{-8}$	2.53 ± 0.07	1.52 ± 0.07	46.83 ± 2.23
Delta	$1.57\times10^{-5} \pm 1.7\times10^{-6}$	1.46 ± 0.08	0.88 ± 0.09	123.90 ± 5.48
Omicron	$2.41\times10^{-7} \pm 6.8\times10^{-8}$	9.05 ± 2.26	8.02 ± 2.28	628.01 ± 44.02

Step B4 (enforce $\mathcal{R}_0$ using expected single-edge transmissibility). Let $L_p^{(m)}$, $L_a^{(m)}$, $L_s^{(m)}$ denote the random discrete-day durations. The expected single-edge transmissibility is

$$T^{(m)}(h_p^{(m)}, h_a^{(m)}, h_s^{(m)}) = 1 - \mathbb{E}\left[e^{-h_p^{(m)} L_p^{(m)}}\right] \left(p_{\text{asym}}^{(m)} \mathbb{E}\left[e^{-h_a^{(m)} L_a^{(m)}}\right] + (1 - p_{\text{asym}}^{(m)}) \mathbb{E}\left[e^{-h_s^{(m)} L_s^{(m)}}\right]\right). \tag{29}$$

We solve for the unique $s > 0$ satisfying

$$T^{(m)}\big(h_p^{(m)}(s), h_a^{(m)}(s), h_s^{(m)}(s)\big) = \frac{\mathcal{R}_0}{\bar{k}}, \tag{30}$$

using bisection.

C. Final epidemic size (FES) matching via synergy exponent in the baseline ABM (optional alignment layer). After $(\beta_p^{(m)}, \beta_a^{(m)}, \beta_s^{(m)})$ are fixed by Steps B1–B4 (thus preserving $\mathcal{R}_0$ and the stage allocation), we optionally tune the synergy exponent η_{syn} in the baseline ABM to match the multi-scale model's mean final epidemic size (FES) for the same variant and $\mathcal{R}_0$. We define FES as the cumulative number of individuals entering I_p over T days, averaged across replicates. A bisection search over η_{syn} is used until the baseline mean FES matches the multi-scale mean FES within a specified tolerance. This additional step reduces residual discrepancies in overall attack rate that can arise because the baseline model uses constant per-stage probabilities whereas the multi-scale model uses time-varying per-edge hazards.

For each variant m and each $\mathcal{R}_0 \in \{1.2, 1.5, 2.0, 2.5\}$, we run 100 stochastic replicates and record daily compartment trajectories, daily counts of newly entering I_p, secondary infection counts, and transmission pairs (day, infector, infectee).

B. Validation of Viral-Kinetics Inference and Multi-Scale Model Outputs

All viral-load trajectories used in this study are based on real longitudinal RT-qPCR Ct data (not synthetic), obtained from the publicly available dataset of Hay et al. [1]. Details of preprocessing (inter-assay calibration, LOD left-censoring, and inclusion criteria) are provided in Section A and the main Methods. Model parameters are calibrated in a modular manner: within-host kinetics (b, γ, δ, V_0) are estimated from the Ct data and then used as fixed inputs to the multi-scale simulations; key between-host epidemiological parameters (stage durations and branching probabilities) are fixed from the literature; and the remaining transmission scale is calibrated to match a target basic reproduction number $\mathcal{R}_0$ under a shared contact network. For variant comparisons, all between-host and network-level components are held fixed and only within-host parameters vary across variants.

This section provides supplementary figures and tables that extend the main text by offering additional validation of the within-host inference framework and further comparison of epidemic outcomes across modeling approaches and SARS-CoV-2 variants.

The Ct value dataset analyzed in this study was derived from publicly available sources [1]. Figure B.1 displays the raw observations prior to preprocessing, with individual Ct measurements shown alongside variant-specific mean trajectories. As described in Section 2.1, we applied a standardized preprocessing pipeline to harmonize sampling times, normalize assay differences, and handle left-censoring. The processed trajectories were subsequently used to estimate viral-kinetics parameters through the hierarchical Bayesian fitting procedure detailed in Section 3.1.

We first present the posterior predictive Ct trajectories generated by the hierarchical viral-kinetics model (Figure B.2). These posterior distributions, obtained via Hamiltonian Monte Carlo sampling, correspond to the parameter estimates reported in Table A.2. The resulting posterior envelopes closely follow the observed Ct trajectories across Alpha, Delta, and Omicron infections, indicating that the piecewise Ct formulation described in Equation (14) of the Methods section effectively captures both the proliferation and clearance phases of viral dynamics. Together, these diagnostics confirm that the inference procedure yields biologically consistent viral-load trajectories and provides a robust foundation for downstream integration into the multi-scale transmission model.

To account for time-specific uncertainty in viral-load measurements, we employ a weighting scheme derived from the posterior distribution of the Ct trajectory model. For each time point t, posterior samples of the Ct trajectory parameters are drawn and transformed into viral load via the Ct–to–viral-load mapping, yielding a sample distribution of $\log_{10}$ viral load at time t. The standard deviation of this distribution, denoted by $\mathrm{sd}(t)$, quantifies the uncertainty associated with the inferred viral load at that time point. These uncertainties are incorporated into the within-host ODE fitting procedure through inverse-variance weighting:

$$w(t) = \frac{1}{\mathrm{sd}(t)^2 + \varepsilon}, \tag{31}$$

where $\varepsilon > 0$ is a small constant included for numerical stability. During parameter estimation, residuals between model-predicted and inferred viral loads are multiplied by $\sqrt{w(t)}$, so that observations with higher posterior uncertainty contribute less to the objective function. To further reduce sensitivity to outliers, we minimize a robust soft-ℓ_1 loss rather than a purely quadratic loss.

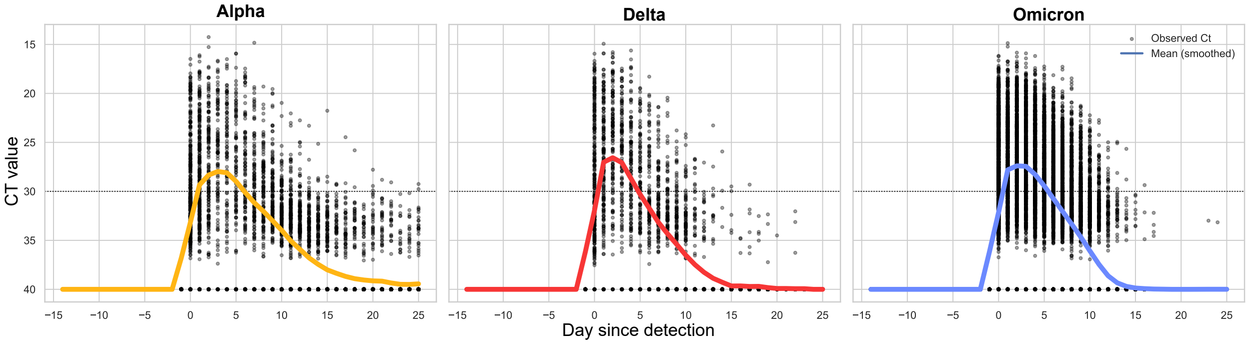

Figure B.1: Observed Ct Values and Variant-Specific Mean Trajectories. Black dots represent the observed Ct values, and each colored curve denotes the mean Ct trajectory for the corresponding SARS-CoV-2 variant.

We then provide epidemic incidence curves across variants and basic reproduction numbers (Figure B.3). For each combination of variant and $\mathcal{R}_0$, we report the time series of newly entering pre-symptomatic individuals (I_p) as a proxy for daily new infections. These plots illustrate how epidemic speed, peak timing, and outbreak magnitude vary systematically with within-host viral kinetics, highlighting the acceleration of epidemic spread under fast-replicating variants such as Omicron relative to Alpha and Delta. They also visualize the widening divergence between baseline and multi-scale transmission dynamics as $\mathcal{R}_0$ increases.

Finally, Table B.1 summarizes key epidemic outcomes—including final epidemic size, peak daily incidence, and peak timing—computed across 100 stochastic simulations for both the baseline (constant transmissibility) and multi-scale (viral-load–modulated) models. By reporting numerical values across all $\mathcal{R}_0$ settings for each variant, the table provides a comprehensive quantitative benchmark that reinforces the main-text findings: the inclusion of time-varying infectiousness produces consistently larger peak sizes, shifts epidemic timing in variant-specific ways, and increases cumulative infections relative to the constant-rate baseline model.

Together, these supplementary materials corroborate the modeling framework and strengthen the empirical support for our conclusions. They demonstrate that the proposed multi-scale approach robustly captures biological variation in viral-load dynamics and translates it into distinct epidemic behaviors across variants and transmission intensities.

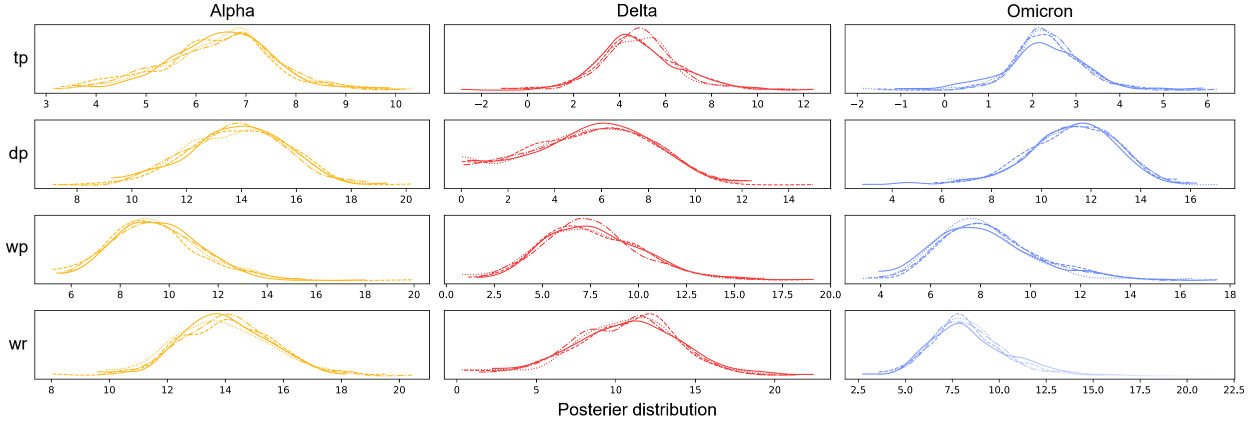

Figure B.2: Posterior predictive Ct trajectories for each SARS-CoV-2 variant. The figure presents the posterior distributions obtained when estimating the Ct trajectory parameters shown in Table 1a. These values are inferred through a sampling process using MCMC, based on the piecewise function in equation (14).

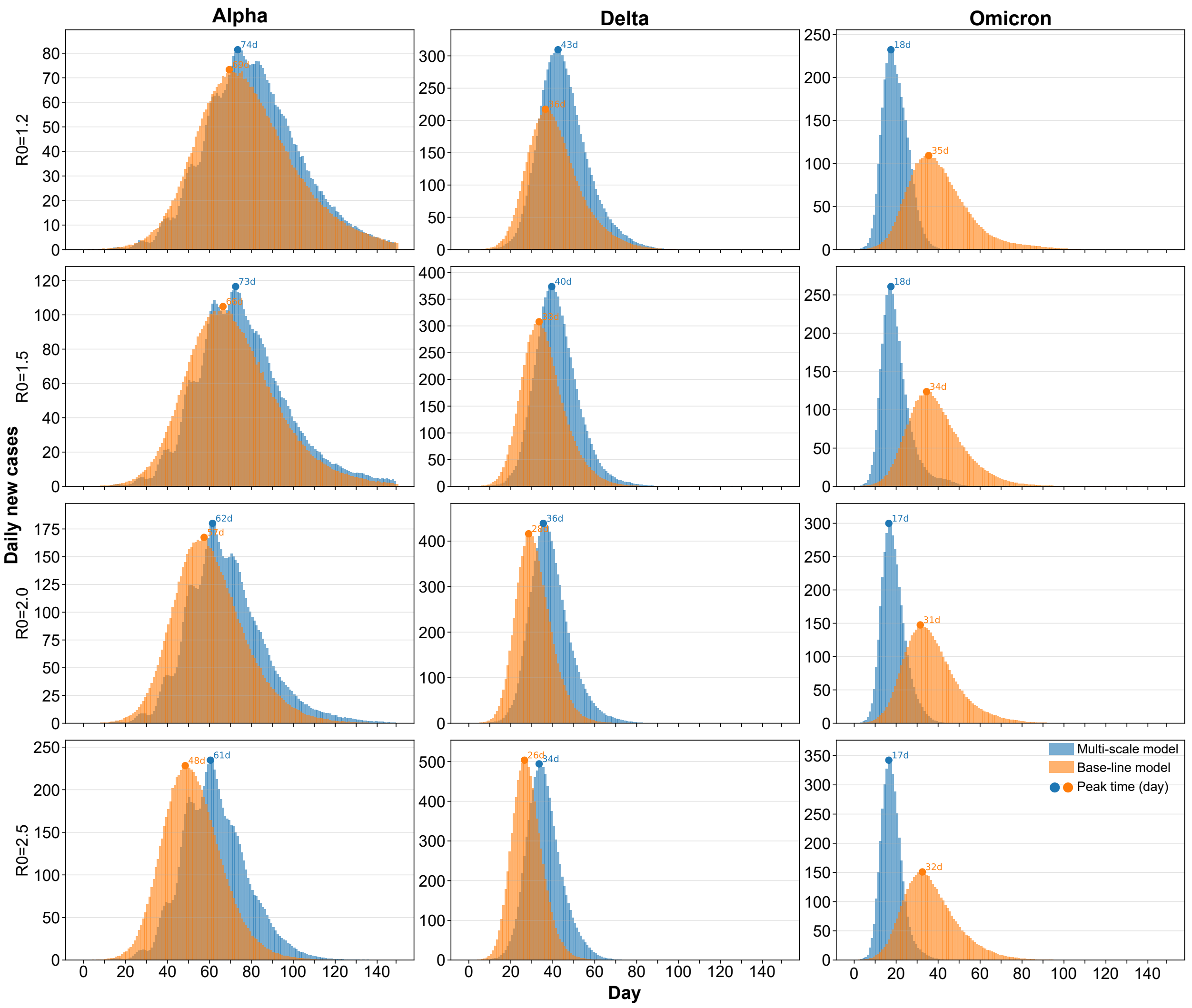

Figure B.3: Daily New Infection Dynamics Across SARS-CoV-2 Variants and $\mathcal{R}_0$ Values. Daily new cases were obtained by counting the number of newly generated pre-symptomatic (I_p) infections each day. Each column corresponds to one of the SARS-CoV-2 variants—Alpha, Delta, and Omicron—while each row represents simulation results under different basic reproduction numbers ($\mathcal{R}_0$) ranging from 1.2 to 2.5.

Table B.1
Epidemic outcomes under baseline (BL; constant transmissibility) and multiscale (MS; viral-load–driven) models by variant and $\mathcal{R}_0$. Entries report mean ± SD across 100 stochastic realizations. "Final size" is cumulative infections (per 10,000), "Peak size" is the maximum daily incidence, and "Peak time" is the day of peak incidence.

Variant	$\mathcal{R}_0$	Model type	Final size	Peak size	Peak time
**Alpha**	1.2	BL	4621.2 ± 158.2	121.8 ± 6.7	72.9 ± 15.0
		MS	4916.1 ± 162.7	135.6 ± 11.4	77.1 ± 12.4
	1.5	BL	5537.5 ± 138.0	160.5 ± 7.6	67.4 ± 13.9
		MS	5969.3 ± 312.8	180.1 ± 12.7	73.1 ± 14.9
	2.0	BL	6752.1 ± 67.4	223.2 ± 9.7	56.3 ± 9.0
		MS	7243.7 ± 68.0	256.6 ± 18.2	66.0 ± 11.0
	2.5	BL	7776.3 ± 56.5	297.6 ± 12.4	48.9 ± 7.0
		MS	8022.1 ± 61.0	315.3 ± 21.2	60.4 ± 9.2
**Delta**	1.2	BL	6683.4 ± 73.1	317.2 ± 12.3	38.3 ± 8.6
		MS	8717.8 ± 59.1	441.7 ± 19.5	44.3 ± 7.8
	1.5	BL	7616.0 ± 62.3	414.5 ± 16.4	33.1 ± 6.0
		MS	9117.6 ± 51.0	511.6 ± 21.5	41.4 ± 6.6
	2.0	BL	8546.5 ± 39.7	541.3 ± 17.8	28.9 ± 4.6
		MS	9478.4 ± 31.8	585.5 ± 21.1	38.6 ± 6.6
	2.5	BL	9071.1 ± 37.1	645.7 ± 19.1	26.0 ± 3.8
		MS	9658.8 ± 23.5	645.2 ± 23.0	35.9 ± 5.4
**Omicron**	1.2	BL	4551.5 ± 131.2	198.8 ± 13.3	36.6 ± 9.9
		MS	8129.0 ± 139.6	836.9 ± 63.9	19.5 ± 4.0
	1.5	BL	4678.7 ± 133.1	205.7 ± 19.0	34.9 ± 7.4
		MS	8363.0 ± 124.3	877.4 ± 55.3	19.7 ± 6.1
	2.0	BL	5197.3 ± 121.6	250.5 ± 16.4	32.6 ± 8.0
		MS	8638.5 ± 115.3	939.6 ± 53.8	18.4 ± 3.9
	2.5	BL	5409.4 ± 130.0	264.6 ± 19.3	33.1 ± 8.1
		MS	8815.7 ± 77.7	992.1 ± 48.7	17.7 ± 3.4

C. Variant-Specific Transmission Network Structures and Tail Distributions

This section provides additional visualization and quantitative evidence for the emergence of heterogeneous transmission patterns and super-spreading dynamics in the multi-scale model. We first present complementary cumulative distribution functions (CCDFs) of secondary infections across variants and reproduction numbers (Figure C.1). These distributions compare the tail behavior of the multi-scale model to the baseline model. Across all variants and $\mathcal{R}_0$ values, the multi-scale formulation produces heavier upper tails, indicating a higher probability of large transmission events and aligning more closely with the characteristic overdispersed behavior observed in empirical SARS-CoV-2 transmission. The inclusion of the reference x^{-2} slope further highlights the stronger tail heaviness and increased super-spreader likelihood under time-varying viral kinetics.

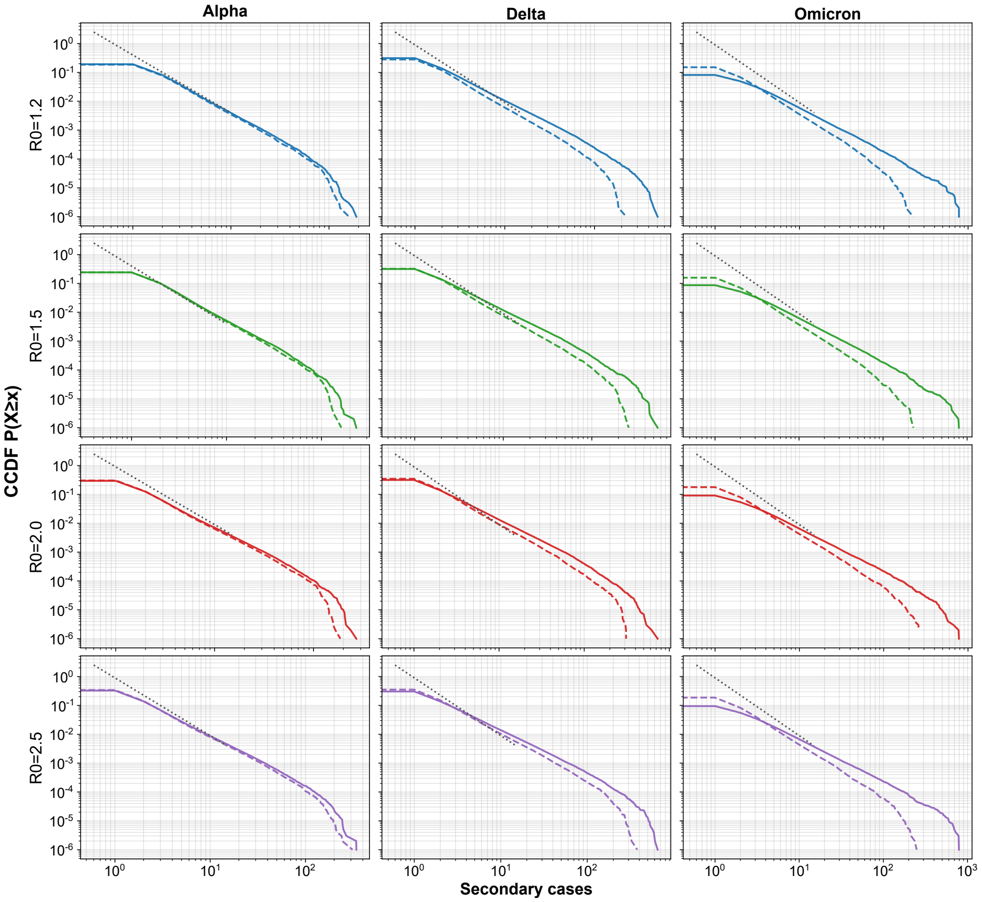

Figure C.1: Complementary cumulative distribution function (CCDF) of secondary cases by variant and $\mathcal{R}_0$. This figure shows the CCDF of secondary infections generated in each simulation scenario. Columns correspond to SARS-CoV-2 variants, and rows represent basic reproduction numbers $\mathcal{R}_0$. Solid curves denote results from the multi-scale model, while dashed curves represent the base-line model.

For reference, an $x^{-2.0}$ line is included to facilitate comparison: curves that lie closer to this reference indicate a higher likelihood of super-spreader events.

We then provide detailed visualizations of reconstructed transmission networks under both modeling frameworks. Figure C.2 shows representative transmission networks generated by the multi-scale model, while Figure C.3 displays analogous networks produced by the baseline model. Each network panel corresponds to a specific combination of variant and $\mathcal{R}_0$, and node coloring reflects the number of secondary infections generated by each individual. These visual comparisons underscore that the multi-scale model consistently yields a greater presence of high-impact transmitters—individuals responsible for disproportionately large numbers of secondary cases—whereas the

baseline model produces more homogeneous transmission patterns. Together, these results illustrate how time-varying infectiousness amplifies transmission heterogeneity and mechanistically generates super-spreading behavior observed in real SARS-CoV-2 epidemics.

To investigate transmission heterogeneity and superspreading structures across variants and reproduction numbers, we reconstructed directed transmission networks from simulation outputs. Each infection event was recorded as a pair (infector-infectee), enabling us to represent individuals as nodes and transmission events as directed edges. Because large-scale simulations generate numerous small transmission fragments, we retained only the largest weakly connected component of each run to highlight the main epidemic cluster. When node counts exceeded the visualization threshold, we further restricted the display to individuals with the highest out-degree (i.e., most secondary infections), preserving the dominant transmission backbone while reducing visual clutter from low-degree peripheral nodes.

Node aesthetics encode transmission burden: node color and size scale with out-degree, with the latter transformed logarithmically and clipped to maintain visibility while emphasizing superspreading nodes. Networks were visualized using a hybrid spring–circular layout, ensuring both hub separation and spatial balance among peripheral nodes. Importantly, these network diagrams are illustrative and were reconstructed from sampled simulation runs to provide qualitative insight into transmission patterns under different SARS-CoV-2 variants and $\mathcal{R}_0$ settings. They should be interpreted as representative visual summaries, rather than exact depictions of every simulated transmission path.

Nodes are colored according to the number of secondary infections they generate, with red nodes indicating individuals with greater impact, highlighting key contributors to secondary transmission.

References

[1] J. A. Hay, S. M. Kissler, J. R. Fauver, C. Mack, C. G. Tai, R. M. Samant, S. Connolly, D. J. Anderson, G. Khullar, M. MacKay, et al., Quantifying the impact of immune history and variant on sars-cov-2 viral kinetics and infection rebound: A retrospective cohort study, Elife 11 (2022) e81849.

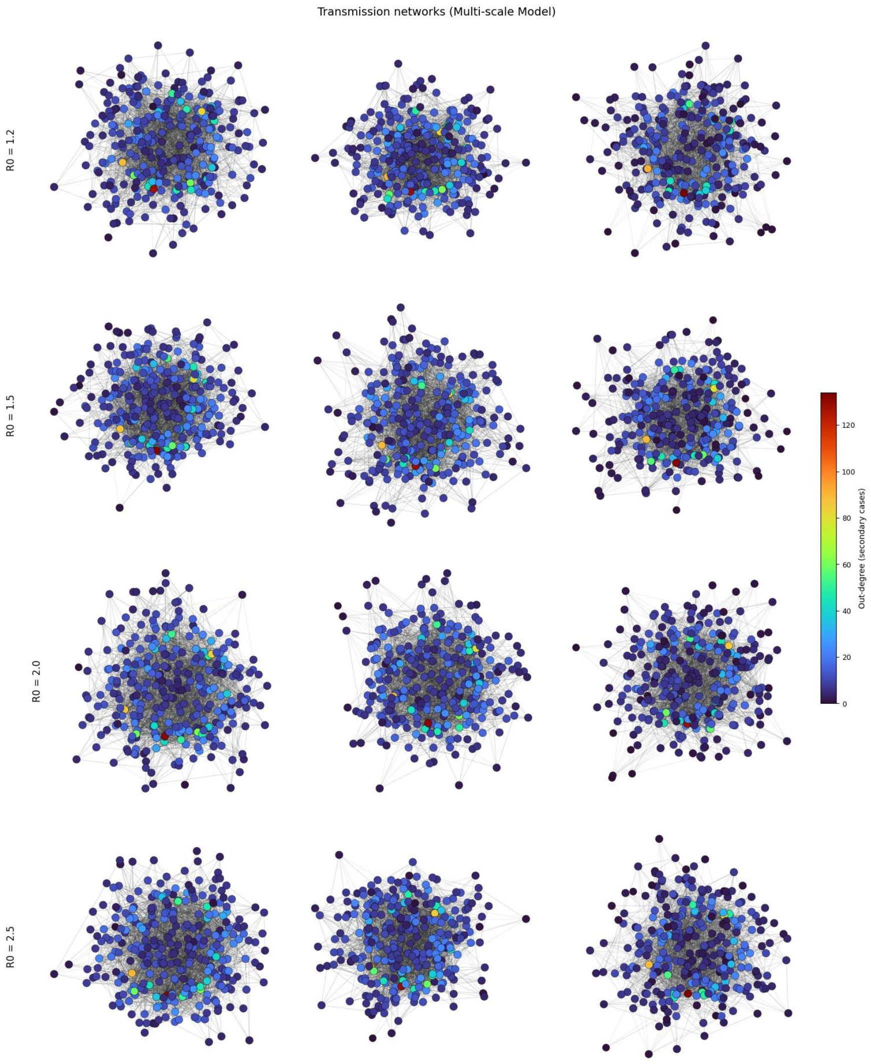

Figure C.2: Transmission Networks Across Variants and $\mathcal{R}_0$ in the Multi-Scale Model. The figure shows a transmission network from the multi-scale model based on transmission pairs. This represents a subset of the reconstructed transmission networks obtained by tracing transmission pairs in each scenario. It extends part of the networks shown in Figure 5, where each row corresponds to $\mathcal{R}_0 = 1.2, 1.5, 2.0,$ and 2.5, and each column corresponds to the Alpha, Delta, and Omicron variants.

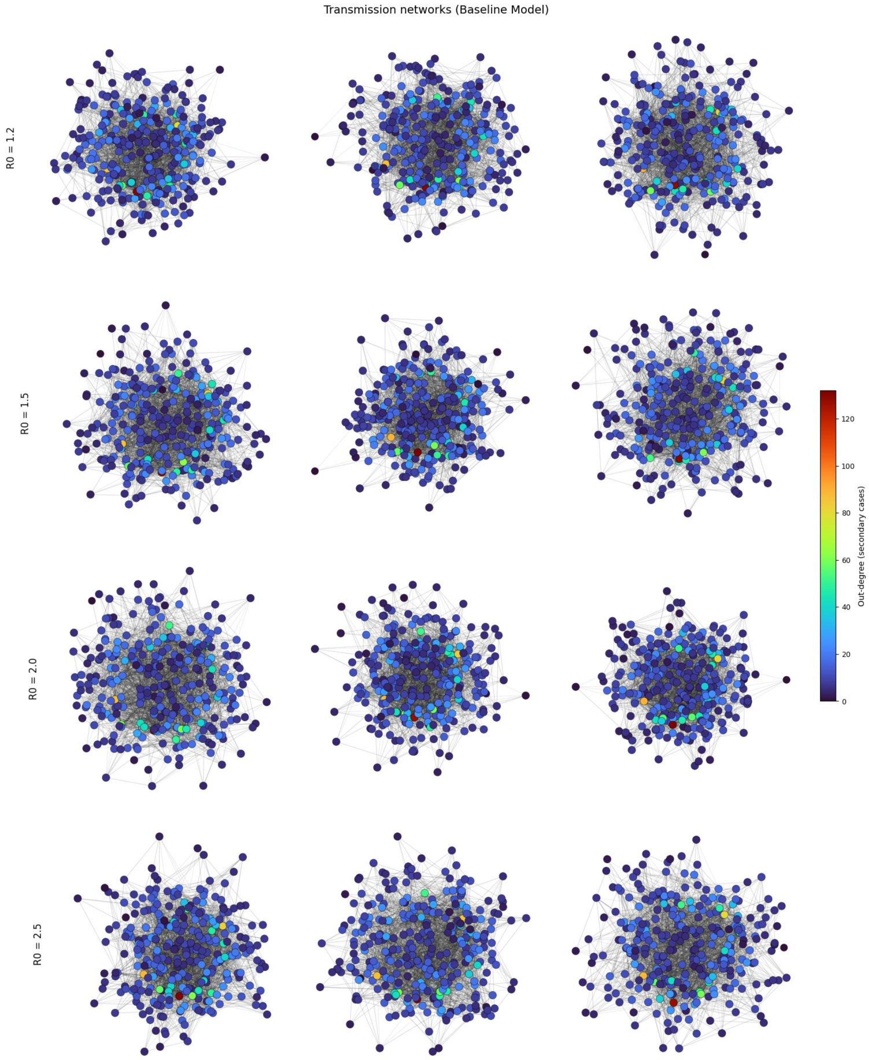

Figure C.3: Transmission Networks Across Variants and $\mathcal{R}_0$ in the Base-line Model. When constructing the network based on transmission pairs, this figure shows the network derived from the base-line scenario. It is presented using the same method as in Figure 5, C.2. where each row corresponds to results for $\mathcal{R}_0 = 1.2, 1.5, 2.0$, and 2.5, and each column represents the Alpha, Delta, and Omicron variants. As before, node colors reflect the number of secondary infections generated, with nodes shaded closer to red indicating individuals with greater impact.
